# Supplementary material for: Molecular adaptation to neoadjuvant immunotherapy in triple-negative breast cancer
Source: Cell Rep Med. 2024 Nov 19;5(11):101825. doi: 10.1016/j.xcrm.2024.101825 (PMC11604547; doi:10.1016/j.xcrm.2024.101825)
Supplement: Document S1. Figures S1–S8 and Tables S1 and S2 [file mmc1.pdf]

Cell Reports Medicine, Volume *5*

Supplemental information

Molecular adaptation to neoadjuvant immunotherapy in triple-negative breast cancer

Carsten Denkert, Andreas Schneeweiss, Julia Rey, Thomas Karn, Akira Hattesohl, Karsten E. Weber, Sivaramakrishna Rachakonda, Michael Braun, Jens Huober, Paul Jank, Hans-Peter Sinn, Dirk-Michael Zahm, Bärbel Felder, Claus Hanusch, Julia Teply-Szymanski, Frederik Marmé, Tanja Fehm, Jörg Thomalla, Bruno V. Sinn, Thorsten Stiewe, Michal Marczyk, Jens-Uwe Blohmer, Marion van Mackelenbergh, Christian Schem, Peter Staib, Theresa Link, Volkmar Müller, Elmar Stickeler, Daniel G. Stover, Christine Solbach, Otto Metzger-Filho, Christian Jackisch, Charles E. Geyer Jr., Peter A. Fasching, Lajos Pusztai, Valentina Nekljudova, Michael Untch, and Sibylle Loibl

Molecular adaptation to neoadjuvant immunotherapy in triple-negative breast cancer

Denkert et al.

Supplemental data

Table S1: Description of the baseline characteristics of the GeparNuevo biomarker cohort (n=148). Related to Figure 1.

Parameter	*Category*	*Durvalumab N=77 N(%)*	*Placebo N=71 N(%)*	*Overall N=148 N(%)*	*p-value*
Age, years	<40	24 (31.2)	16 (22.5)	40 (27.0)	0.237
	>=40	53 (68.8)	55 (77.5)	108 (73.0)	
Window arm	window	52 (67.5)	44 (62.0)	96 (64.9)	0.496
	no window	25 (32.5)	27 (38.0)	52 (35.1)	
Menopausal Status	premenopausal	45 (58.4)	41 (57.7)	86 (58.1)	1.000
	postmenopausal	32 (41.6)	30 (42.3)	62 (41.9)	
cT	cT1-2	70 (90.9)	68 (95.8)	138 (93.2)	0.239
	cT3-4	7 (9.1)	3 (4.2)	10 (6.8)	
cN by sonography	cN0	51 (67.1)	50 (70.4)	101 (68.7)	0.665
	cN+	25 (32.9)	21 (29.6)	46 (31.3)	
	missing	1	0	1	
Breast Cancer Stage (binary)	Stage 0 or I	27 (35.1)	26 (36.6)	53 (35.8)	0.865
	Stage IIA and higher	50 (64.9)	45 (63.4)	95 (64.2)	
Grading	G2	13 (16.9)	13 (18.3)	26 (17.6)	0.820
	G3	64 (83.1)	58 (81.7)	122 (82.4)	
Histological tumor type	ductal or ductal-lobular invasive	60 (77.9)	58 (81.7)	118 (79.7)	0.437
	lobular invasive	0 (0.0)	1 (1.4)	1 (0.7)	
	other	17 (22.1)	12 (16.9)	29 (19.6)	
sTILs	low (0-10%)	27 (35.1)	26 (36.6)	53 (35.8)	0.852
	intermediate (11-59%)	39 (50.6)	33 (46.5)	72 (48.6)	
	high (60-100%)	11 (14.3)	12 (16.9)	23 (15.5)	
PD-L1	negative	8 (11.3)	10 (14.9)	18 (13.0)	0.616
	positive	63 (88.7)	57 (85.1)	120 (87.0)	
	missing	6	4	10	
pCR (ypT0 ypN0)	no	35 (45.5)	35 (49.3)	70 (47.3)	0.742
	yes	42 (54.5)	36 (50.7)	78 (52.7)	

p: Fisher's exact test resp. Pearson's Chi-squared test

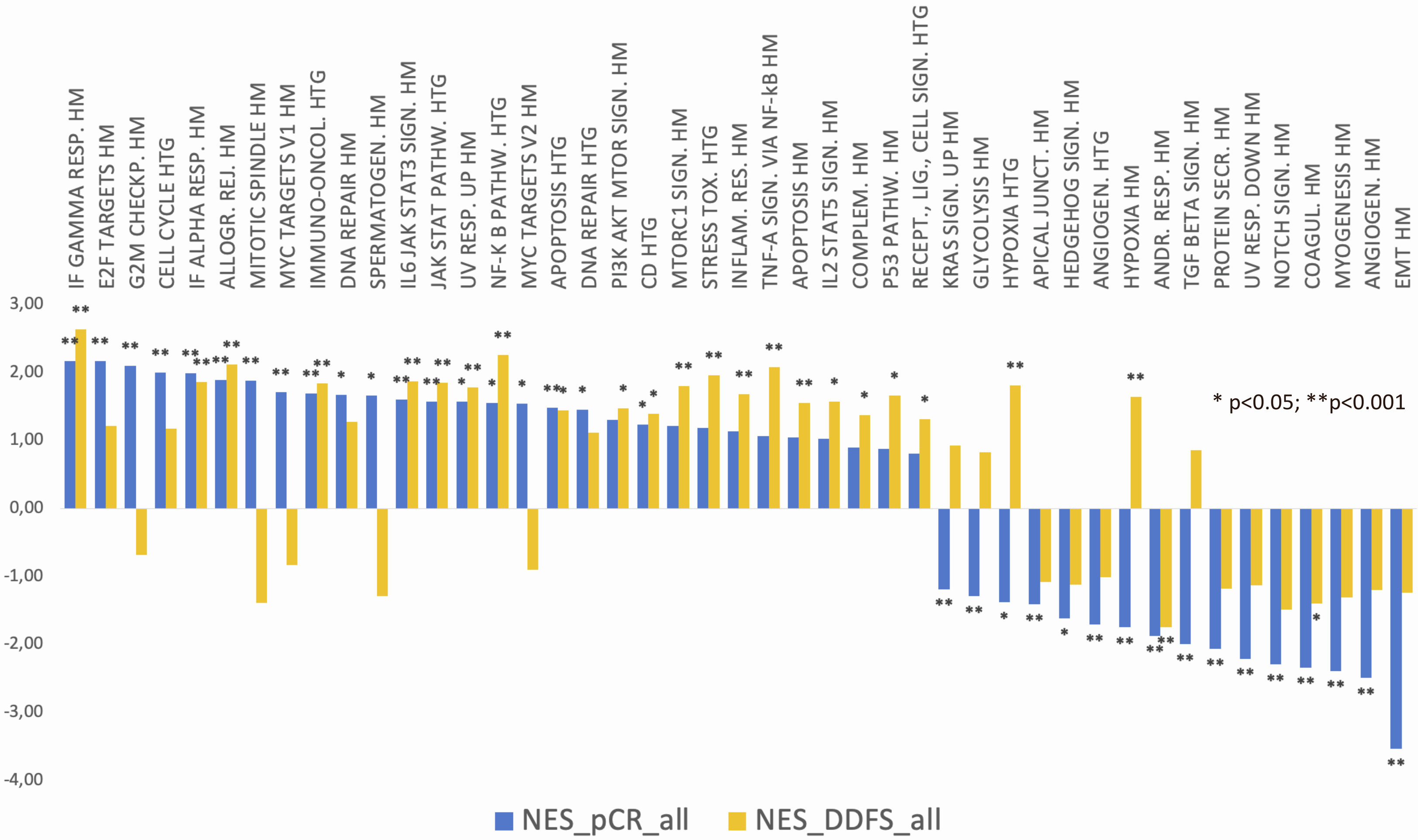

Figure S1: Gene set enrichment analysis for the two clinical endpoints pCR and DDFS (related to figure 2)

Complete G9 cohort: Details of the gene set enrichment analysis based on an analysis of 2549 genes included in the HTG oncology biomarker assay using HTG-defined gene sets (HTG) as well as the Hallmark gene sets (HM). The normalized enrichment score (NES) for pCR (blue) and DDFS (orange) is shown. Only gene sets are included that have a p-value of <0.05 as well as a FDR of <25% for at least one of the endpoints (pCR or DDFS). The analysis shown is based on pretherapeutic core biopsies of the complete cohort, combining both therapy arms.

Complete G9 cohort: genesets for significantly improved DDFS and significantly increased pCR rate

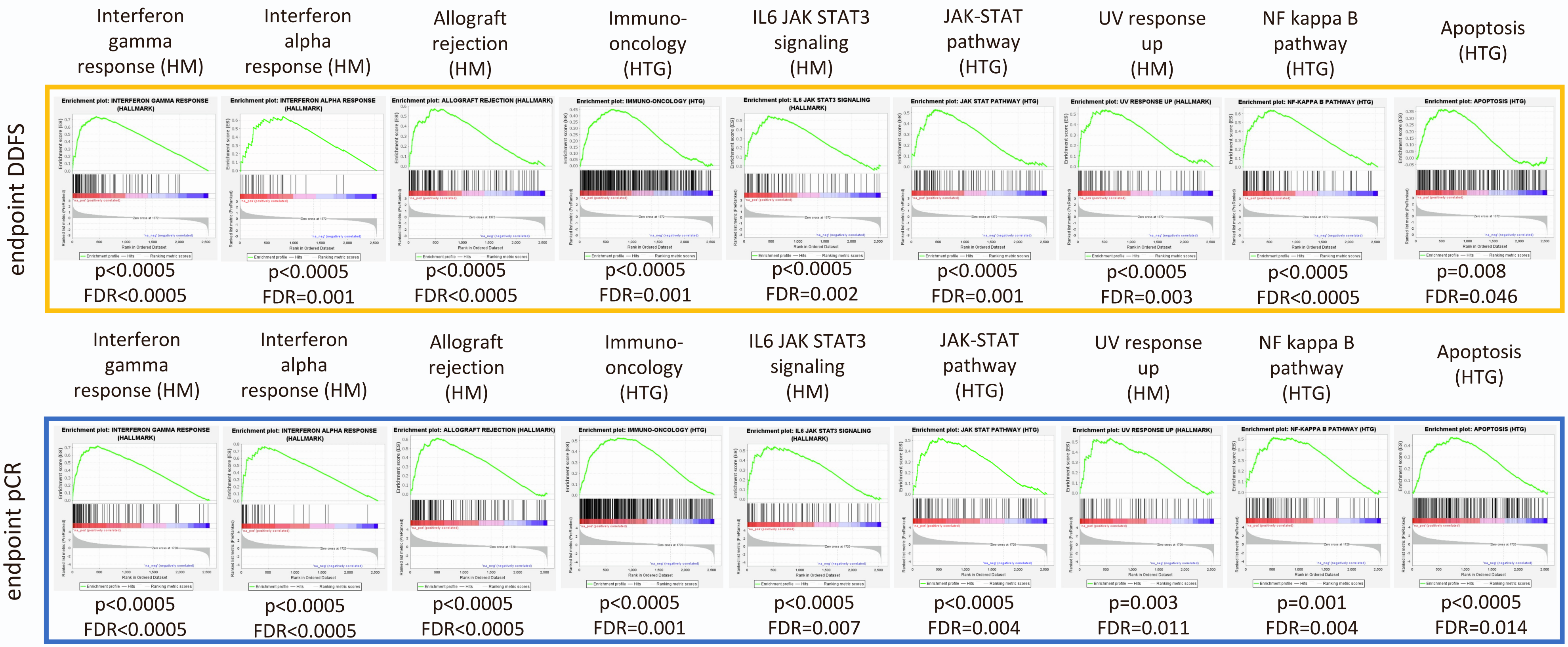

Figure S2: Gene set enrichment analysis for the two clinical endpoints pCR and DDFS (related to figure 2)
Complete G9 cohort: gene sets for significantly improved DDFS and significantly increased pCR rate.

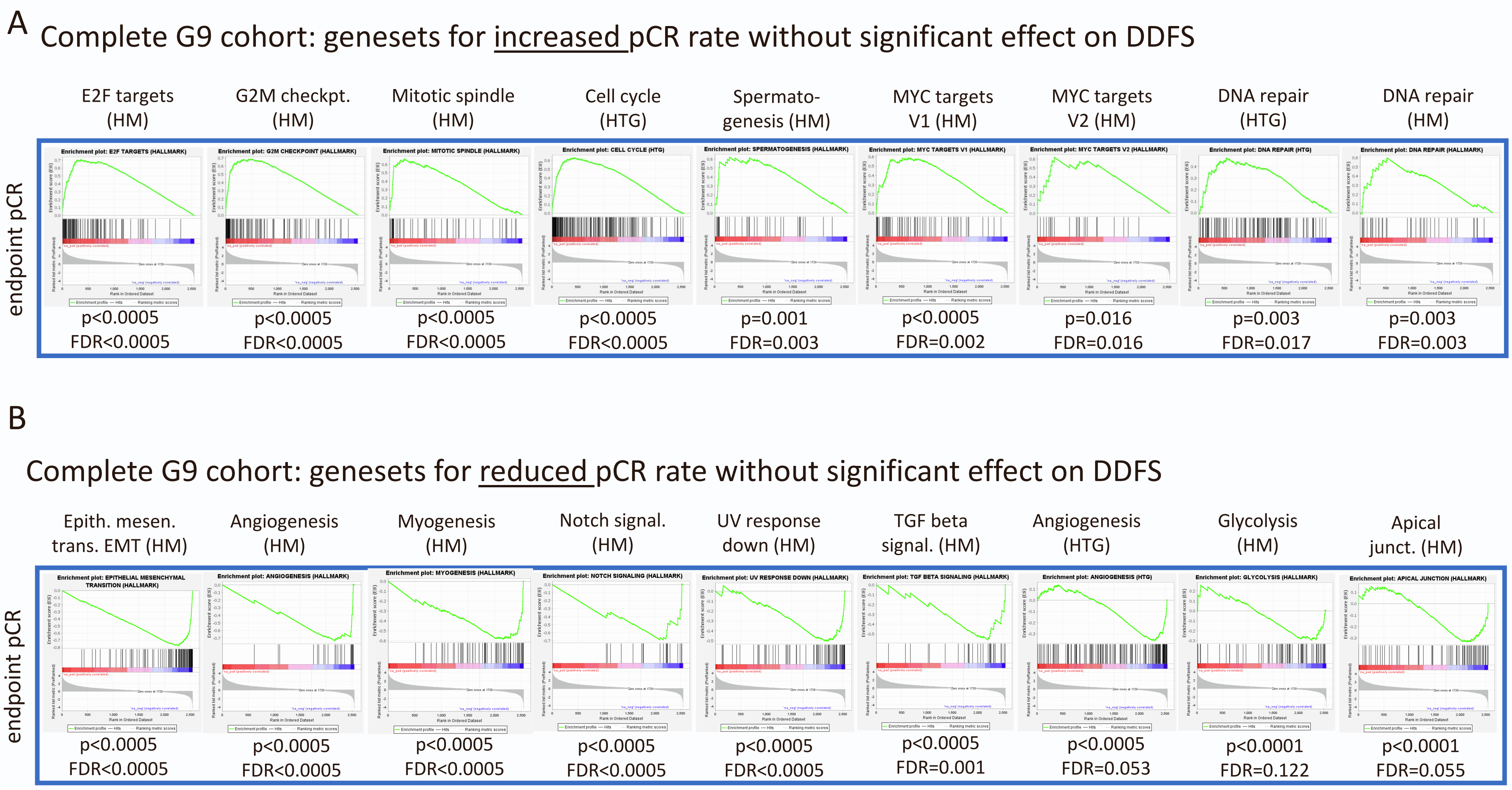

Figure S3: Gene set enrichment analysis for the two clinical endpoints pCR and DDFS (related to figure 2)
(A) Complete G9 cohort: gene sets for increased pCR rate without significant effect on DDFS.
(B) Complete G9 cohort: gene sets for reduced pCR rate without significant effect on DDFS.

Denkert et al.

Figure S4

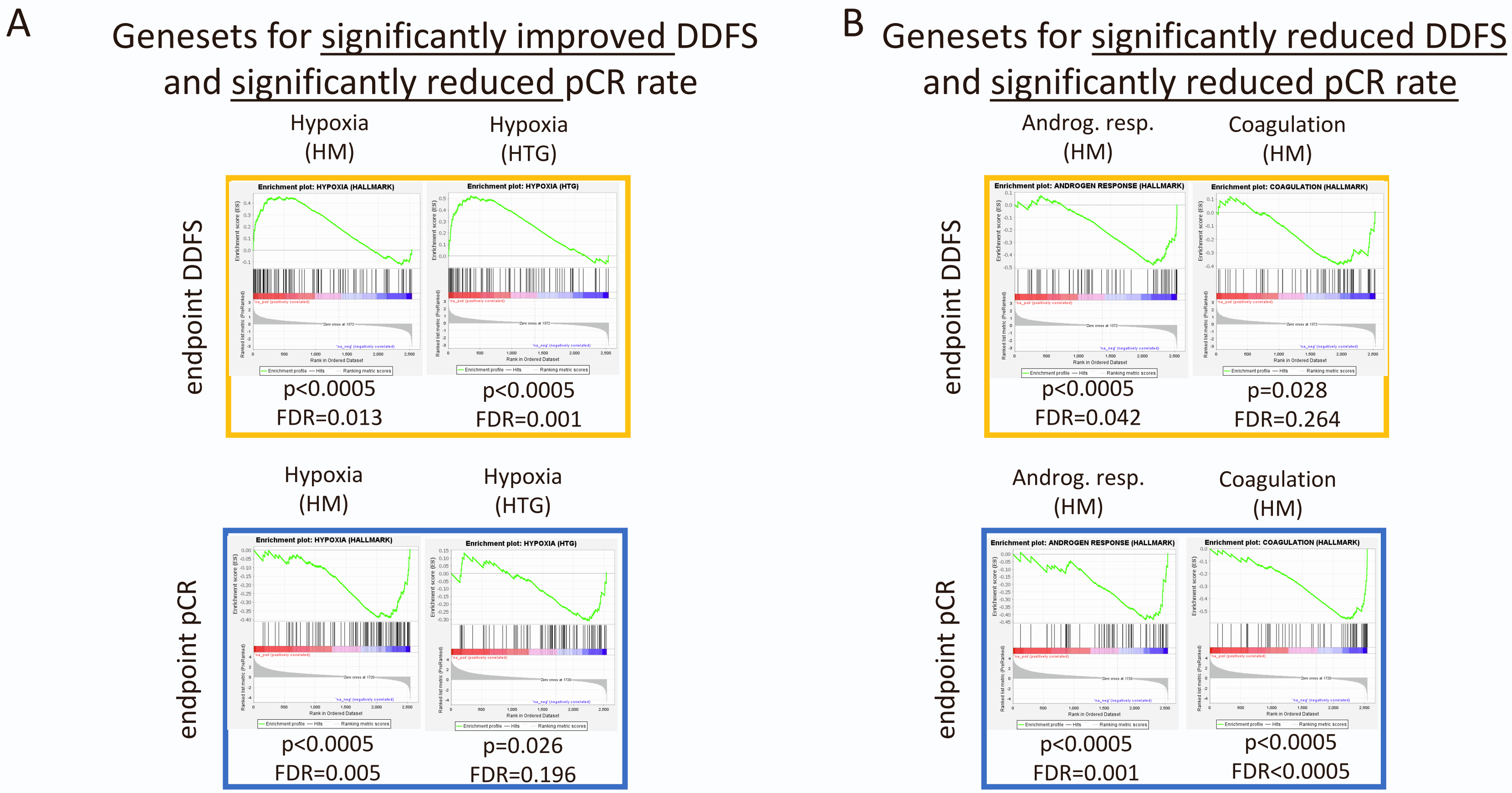

Figure S4: Gene set enrichment analysis for the two clinical endpoints pCR and DDFS (related to figure 2)

(A) Complete G9 cohort: gene sets for significantly improved DDFS and significantly reduced pCR rate.

(B) Complete G9 cohort: gene sets for significantly reduced DDFS and significantly reduced pCR rate.

Denkert et al.

Figure S5

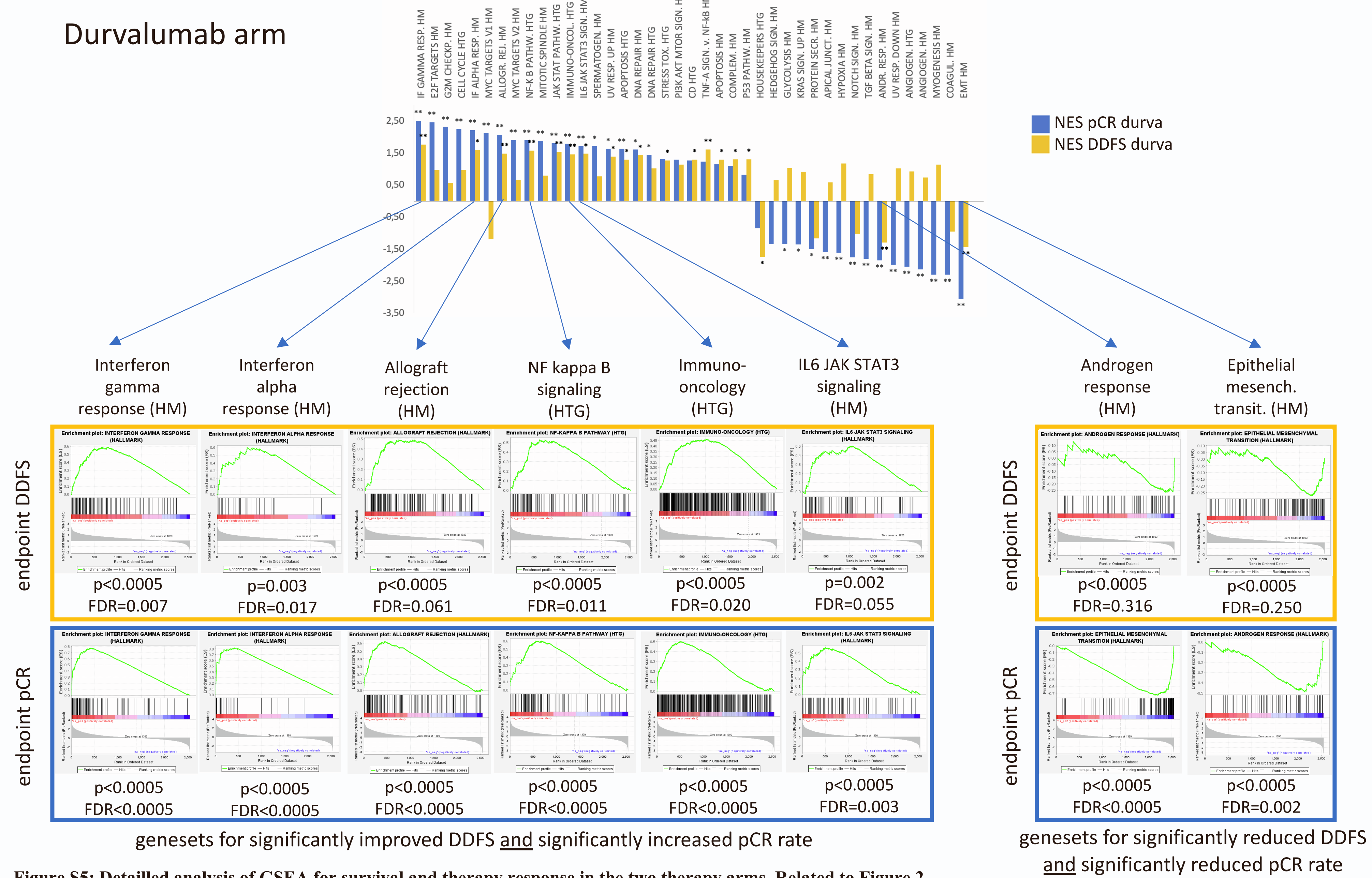

Figure S5: Detailled analysis of GSEA for survival and therapy response in the two therapy arms. Related to Figure 2. Durvalumab arm: Additional details of the analysis shown in Figure 2: GSEA plots for the most important cellular pathways.

Denkert et al.

Figure S6

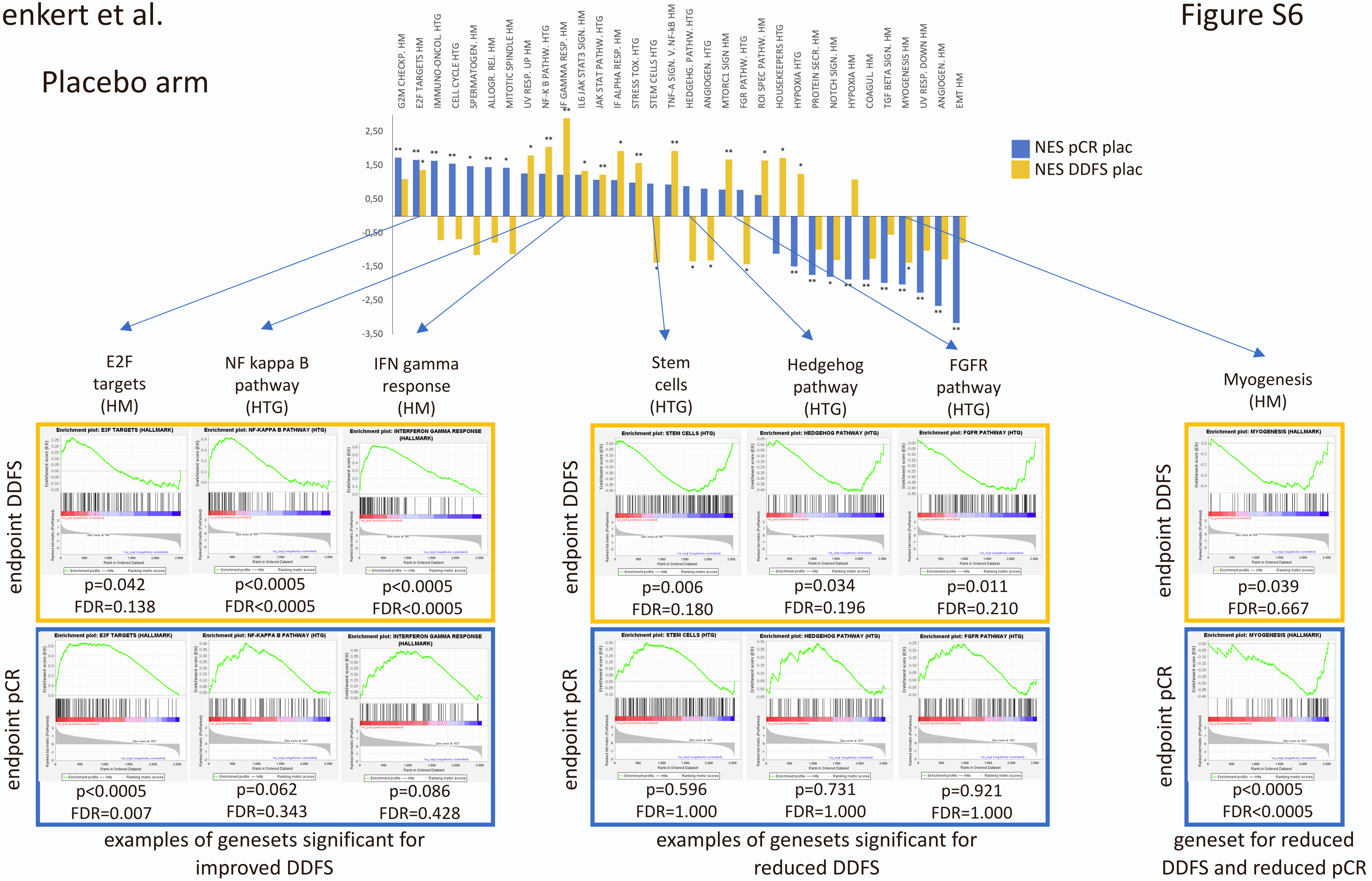

Figure S6: Detailled analysis of GSEA for survival and therapy response in the two therapy arms. Related to Figure 2.
Placebo arm: Additional details of the analysis shown in Figure 2: GSEA plots for the most important cellular pathways.

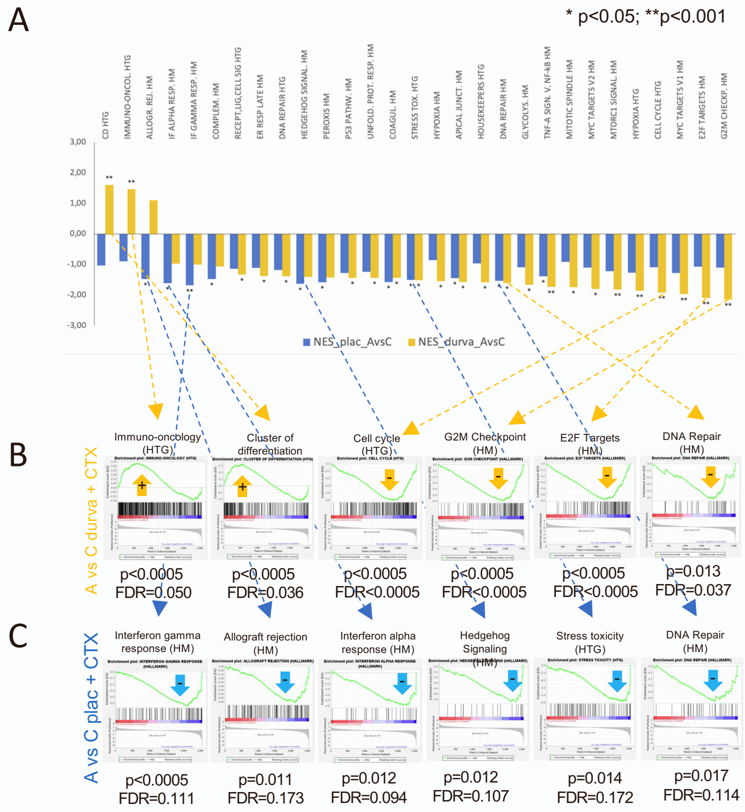

Figure S7: Evaluation of molecular alterations in TNBC before therapy (A-samples) compared with samples after 12 weeks of chemotherapy +/- durvalumab (C-samples). Related to Figure 5.

(**A**) Comparison of gene set enrichment in both therapy arms after 12 weeks of chemotherapy with durvalumab (orange) or without durvalumab (blue). The normalized enrichment score (NES) for placebo (x-axis, blue) and durvalumab (y-axis, orange) is shown.

(B,C) GSEA results for selected gene sets after 12 weeks of chemotherapy with **(B)** or without **(C)** durvalumab. (CTX, chemotherapy; FDR, false discovery rate)

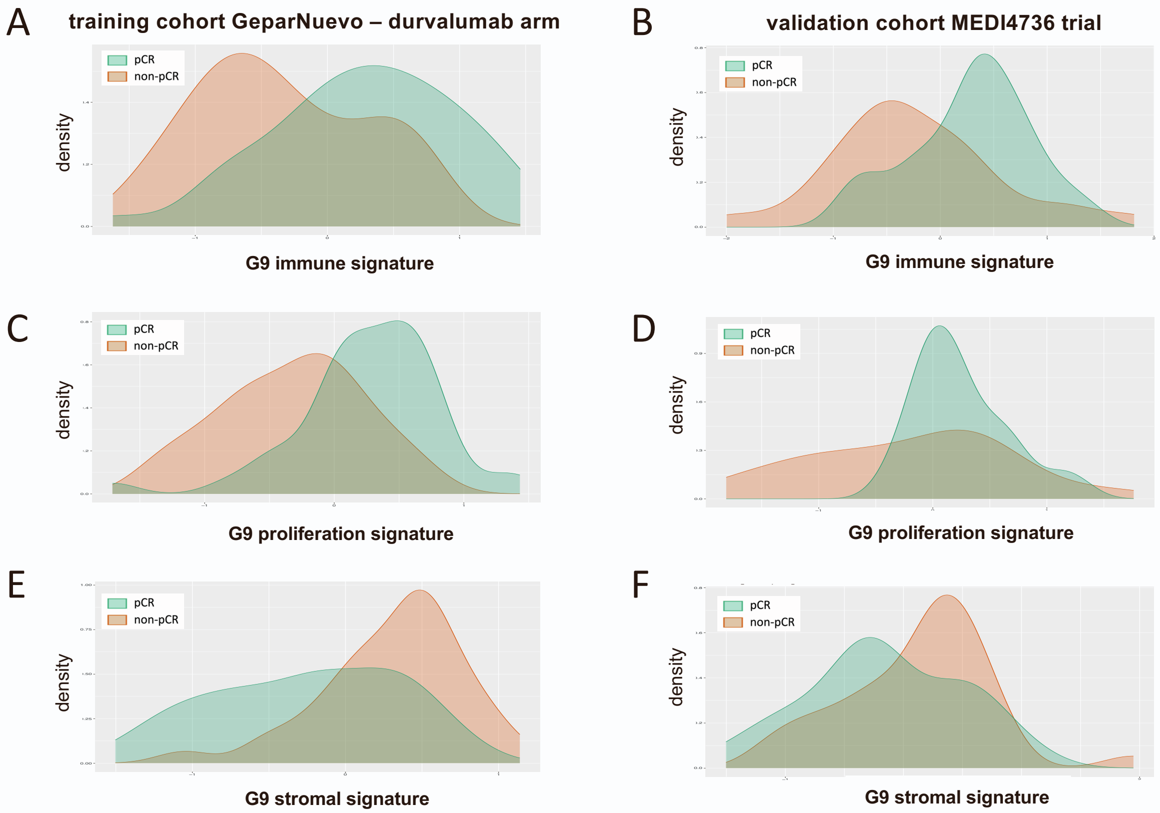

Figure S8: Validation in three independent cohorts (Related to Figure 7). Comparison of distribution of pCR rate for three gene signatures covering immune **(A,B)**, proliferation **(C,D)** and stromal genes **(E,F)** in the GeparNuevo durvalumab training cohort **(A,C,E)** and the validation cohort MEDI4736 **(B,D,F)**.

The negative role of stromal gene expression and the positive role of immune and proliferation genes could be validated in the independent MEDI4736 cohort. For full details see STAR Methods section.

Table S2. Genes included in the stromal, proliferation and immune signature (Related to Figure 7 and Figure S8)

G9.immune.signature.genes	G9.proliferation.signature.genes	G9.stromal.signature.genes
ADAR	ATR	ACTA2
APOL3	AURKA	ANXA1
CA9	AURKB	CALD1
CCL2	BLM	CD55
CCL4	BUB1	COL11A1
CCL5	CASP8AP2	COL1A1
CCL7	CBX5	COL1A2
CD274	CCNA2	COL3A1
CD38	CCNB1	COL5A1
CD47	CCNE1	COL5A2
CD79A	CDC20	COMP
CD86	CDC7	DST
CD8A	CDK2	EDIL3
CDKN2A	CDKN1B	EFNB2
CEBPB	CDKN2A	F3
CHI3L1	CDKN3	FBN1
CTSS	CHEK2	FGF13
CXCL10	CKS2	FLT1
CXCL13	CTPS1	FN1
CXCL9	CUL2	GNG12
CXCR6	DLGAP5	GSN
DDX58	DNMT1	IGFBP4
DHX58	E2F3	IGFBP7
EIF2AK2	ECT2	ITGA2
FGL2	EZH2	ITGAV
GBP1	FBXO5	JAG1
GNLY	GTSE1	KDR
GZMA	HELLS	LOX
GZMB	HIST1H3H	MMP14
HLA-A	HJURP	MMP2
HLA-B	HMGB2	NOTCH4
HLA-E	KIF2C	PCOLCE
ICAM1	KNTC1	PDGFA
IDO1	KPNA2	PDGFRB
IFI27	LIG1	PDLIM7
IFIT2	LIN9	PLAT
IL15	MAD2L1	PMEPA1
IL17RB	MAX	RB1
IL2RA	MCM3	RUNX1
IL2RB	MCM5	SERPINF1
IL2RG	MCM6	SFRP4
IL6R	MELK	SPARC
IRF1	MKI67	SPDEF
IRF2	NASP	THBS1

IRF3
IRF4
IRF7
IRF9
ISG15
ITGB7
JAK2
LAG3
LYZ
MAP4K1
MX1
NFKB1
OAS1
OASL
PDCD1
PDCD1LG2
PML
PRDM1
PRF1
SLAMF7
SOCS1
STAT1
STAT2
TAP1
TAP2
TLR3
TNFAIP3
TRAF2
TYMP
VCAM1

NDC80
NOLC1
NUF2
NUP62
NUSAP1
PA2G4
PLK4
PML
POLE2
PRC1
PSIP1
RACGAP1
RAD9A
RPA1
RUVBL1
SKP2
SMC1A
SMC4
STMN1
TERF1
TFDP1
TOP2A
TOP3A
TP53
TPX2
TTK
UBB
WHSC1

THBS2
TIMP3
VEGFB
VEGFC
