# Supplementary material for: Molecular adaptation to neoadjuvant immunotherapy in triple-negative breast cancer
Source: Cell Rep Med. 2024 Nov 19;5(11):101825. doi: 10.1016/j.xcrm.2024.101825 (PMC11604547; doi:10.1016/j.xcrm.2024.101825)
Supplement: Document S2. Article plus supplemental information [file mmc2.pdf]

Cell Reports Medicine

Article

Molecular adaptation to neoadjuvant immunotherapy in triple-negative breast cancer

Graphical abstract

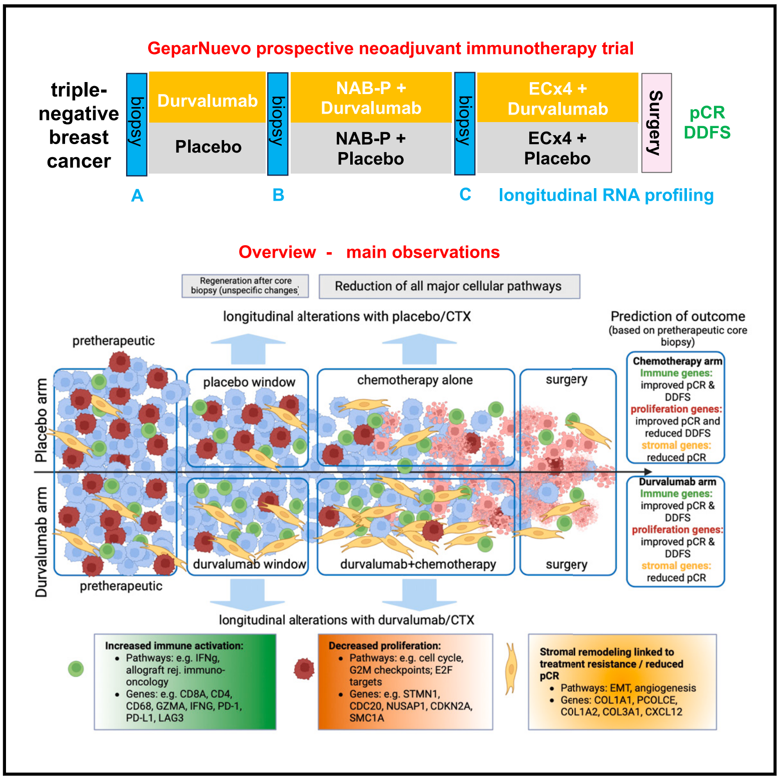

Highlights

- Inhibition of PD-L1 leads to the activation of immune and stromal gene expression
- Immune, proliferation, and stromal gene sets are linked to therapy response and prognosis
- Stromal gene expression is associated with reduced pCR rate and prognosis
- The results are relevant for response prediction and new combination therapy concepts

Authors

Carsten Denkert, Andreas Schneeweiss, Julia Rey, ..., Valentina Nekljudova, Michael Untch, Sibylle Loibl

Correspondence

carsten.denkert@uni-marburg.de (C.D.), sibylle.loibl@gbg.de (S.L.)

In brief

Denkert et al. evaluate the longitudinal changes in tumor tissue of triple-negative breast cancer in a clinical trial cohort treated with neoadjuvant immunotherapy plus chemotherapy. The role of different gene sets in the prediction of therapy response and prognosis was analyzed, with a focus on immune, proliferation, and stromal genes.

Denkert et al., 2024, Cell Reports Medicine 5, 101825
November 19, 2024 © 2024 The Authors. Published by Elsevier Inc.
https://doi.org/10.1016/j.xcrm.2024.101825

CellPress

Cell Reports Medicine

CellPress
OPEN ACCESS

Article

Molecular adaptation to neoadjuvant immunotherapy in triple-negative breast cancer

Carsten Denkert,[1,*] Andreas Schneeweiss,[2] Julia Rey,[3] Thomas Karn,[4] Akira Hattesohl,[1] Karsten E. Weber,[3] Sivaramakrishna Rachakonda,[3] Michael Braun,[5] Jens Huober,[6] Paul Jank,[1] Hans-Peter Sinn,[7] Dirk-Michael Zahm,[8] Bärbel Felder,[3] Claus Hanusch,[5] Julia Teply-Szymanski,[1] Frederik Marmé,[9] Tanja Fehm,[10]

(Author list continued on next page)

[1]Institute of Pathology, Philipps University Marburg, Marburg University Hospital (UKGM), and University Cancer Center Frankfurt-Marburg (UCT), Marburg, Germany
[2]Nationales Centrum für Tumorerkrankungen, Universitätsklinikum und Deutsches Krebsforschungszentrum, Heidelberg, Germany
[3]German Breast Group (GBG) Forschungs GmbH, Neu-Isenburg, Germany
[4]University Hospital, Goethe University, and University Cancer Center Frankfurt-Marburg (UCT), Frankfurt am Main, Germany
[5]Rotkreuzklinikum München, Munich, Germany
[6]Kantonsspital St. Gallen, Brustzentrum, St. Gallen, Switzerland
[7]Institut für Pathologie, Universitätsklinikum Heidelberg, Heidelberg, Germany
[8]SRH Waldklinikum Gera GmbH, Gera, Germany
[9]Medizinische Fakultät Mannheim, Universität Heidelberg, Universitätsfrauenklinik Mannheim, Mannheim, Germany
[10]Frauenklinik, Universitätsklinikum Düsseldorf, Center for Integrated Oncology (CIO Aachen, Bonn, Cologne, Düsseldorf), Düsseldorf, Germany
[11]Praxis für Hämatologie und Onkologie Koblenz, Germany
[12]Institut für Pathologie, Charité Universitätsmedizin Berlin, Berlin, Germany
[13]Institute of Molecular Oncology and Genomics Core Facility, Member of the German Center for Lung Research (DZL), Philipps University Marburg, Marburg, Germany
[14]Institute of Lung Health (ILH), Justus Liebig University, Giessen, Germany
[15]Department of Data Science and Engineering, Silesian University of Technology, Gliwice, Poland
[16]Yale School of Medicine, New Haven, CT, USA
[17]Klinik für Gynäkologie mit Brustzentrum, Charité-Universitätsmedizin Berlin, Berlin, Germany
[18]Klinik für Gynäkologie und Geburtshilfe, Universitätsklinikum Schleswig-Holstein, Kiel, Germany
[19]Mammazentrum Hamburg, Brustzentrum am Krankenhaus Jerusalem, Hamburg, Germany
[20]Klinik für Hämatologie und Onkologie, St.-Antonius Hospital, Eschweiler, Germany
[21]Klinik und Poliklinik für Frauenheilkunde und Geburtshilfe, Universitätsklinikum Carl Gustav Carus Dresden, Dresden, Germany

(Affiliations continued on next page)

SUMMARY

Therapy-induced molecular adaptation of triple-negative breast cancer is crucial for immunotherapy response and resistance. We analyze tumor biopsies from three different time points in the randomized neoadjuvant GeparNuevo trial (NCT02685059), evaluating the combination of durvalumab with chemotherapy, for longitudinal alterations of gene expression. Durvalumab induces an activation of immune and stromal gene expression as well as a reduction of proliferation-related gene expression. Immune genes are positive prognostic factors irrespective of treatment, while proliferation genes are positive prognostic factors only in the durvalumab arm. We identify stromal-related gene expression as a contributor to immunotherapy resistance and poor therapy response. The results provide evidence from clinical trial cohorts suggesting a role for stromal reorganization in therapy resistance to immunotherapy and in the generation of an immune-suppressive microenvironment, which might be relevant for future therapy approaches targeting the tumor stroma parallel to immunotherapy, such as combinations of immunotherapy with anti-angiogenic therapy.

INTRODUCTION

Triple-negative breast cancer (TNBC) has a poor prognosis on one hand, but on the other hand, a high response rate to chemotherapy,[1,2] and the responding patients have an excellent prognosis.[3–5] Tumor proliferation is an indicator of poor prognosis, but a predictor of improved chemotherapy response.[6]

Immune checkpoint inhibitor therapy in combination with chemotherapy is standard of care for early[7,8] and metastatic TNBC.[9,10] For a better understanding of the adaptation of the

© 2024 The Authors. Published by Elsevier Inc.
This is an open access article under the CC BY license (http://creativecommons.org/licenses/by/4.0/).

Jörg Thomalla,[11] Bruno V. Sinn,[12] Thorsten Stiewe,[13,14] Michal Marczyk,[15,16] Jens-Uwe Blohmer,[17] Marion van Mackelenbergh,[18] Christian Schem,[19] Peter Staib,[20] Theresa Link,[21] Volkmar Müller,[22] Elmar Stickeler,[23] Daniel G. Stover,[24] Christine Solbach,[25] Otto Metzger-Filho,[26] Christian Jackisch,[27] Charles E. Geyer Jr.[28] Peter A. Fasching,[29] Lajos Pusztai,[30] Valentina Nekljudova,[3] Michael Untch,[31] and Sibylle Loibl[3,32,*]
[22]Klinik und Poliklinik für Gynäkologie, Universitätsklinikum Hamburg Eppendorf, Hamburg, Germany
[23]Department of Obstetrics and Gynecology, Center for Integrated Oncology (CIO Aachen, Bonn, Cologne, Düsseldorf), University Hospital of RWTH Aachen, Aachen, Germany
[24]Department of Internal Medicine, Ohio State University, Columbus, OH, USA
[25]Brustzentrum am Universitätsklinikum Frankfurt, Frankfurt, Germany
[26]Dana-Farber Cancer Institute, Boston, MA, USA
[27]Klinik für Gynäkologie und Geburtshilfe, Sana Klinikum Offenbach, Offenbach, Germany
[28]Department of Medicine, University of Pittsburgh Medical Center, Pittsburgh, PA, USA
[29]Department of Gynecology and Obstetrics, University Hospital Erlangen, Comprehensive Cancer Center Erlangen-Nuremberg, National Center for Tumour Diseases, Erlangen, Germany
[30]Yale Cancer Center, Yale School of Medicine, New Haven, CT, USA
[31]Department of Gynecology and Obstetrics, Breast Cancer Center, Helios Klinikum Berlin Buch, Berlin, Germany
[32]Lead contact
*Correspondence: carsten.denkert@uni-marburg.de (C.D.), sibylle.loibl@gbg.de (S.L.)
https://doi.org/10.1016/j.xcrm.2024.101825

tumor microenvironment to immunotherapy, it is important to separate the molecular effects of immunotherapy from those of conventional chemotherapy and to identify cellular pathways that are in control of therapy response and resistance as well as long-term outcome.

In the neoadjuvant setting, patients with pathological complete response (pCR) have an excellent outcome.[11] However, on the clinical trial level, an increased pCR rate does not always result in an improved overall survival. On the other hand, improved survival outcome could be observed also in neoadjuvant clinical trials without significant improvements in pCR rate.[12,13]

In this translational research program, we analyzed a total of 247 biopsies taken at three different time points during the GeparNuevo trial (NCT02685059),[14] a neoadjuvant trial evaluating the addition of durvalumab to chemotherapy. The GeparNuevo clinical design is particularly suitable for these investigations, because in the window phase of this trial, a subset of patients received one dose of durvalumab vs. placebo before the addition of chemotherapy 2 weeks later. Therefore, it is possible to separate the molecular alterations induced by durvalumab alone from complex alterations induced by the durvalumab plus chemotherapy combination.

The overall aim was the identification of pathways and genes relevant for therapy resistance and survival after durvalumab, and the dissection of longitudinal molecular alterations induced by durvalumab. The results were validated in independent clinical cohorts, including the MEDI4736 trial (NCT02489448)[15,16] investigating the neoadjuvant durvalumab-chemotherapy combination in TNBC, the BrighTNess trial (NCT02032277)[17,18] investigating a neoadjuvant combination therapy approach without an immune checkpoint inhibitor, as well as the SCAN-B (survival data with non-immune therapy, NCT02306096) cohort.

RESULTS

Biomarker cohort and longitudinal samples

Clinical trial design and samples for biomarker analysis are shown in Figure 1. A total of 247 tumor samples were evaluated by gene expression analysis: 148 pretherapeutic core biopsies (A-samples; durvalumab: n = 77; placebo: n = 71), 72 samples after the window phase (B-samples), and 27 samples after 12 weeks of nab-paclitaxel therapy (C-samples). Table S1 shows the clinicopathological parameters of the GeparNuevo biomarker cohort (n = 148). The endpoints pCR (no residual tumor in breast and lymph nodes after neoadjuvant therapy) and distant disease-free survival (DDFS) were evaluated.

Gene signatures relevant for pCR and/or DDFS in the complete cohort

One of the main clinical results of the GeparNuevo trial was the observation of a significant survival benefit,[14] despite only a moderate, non-significant increase in pCR rate.[12] In the biomarker cohort used for this translational investigation, the main results of the clinical trial were still evident, with a non-significant increased pCR rate with neoadjuvant durvalumab (54.5%) compared to placebo (50.7%) as well as a significantly improved DDFS with neoadjuvant durvalumab compared with placebo (stratified hazard ratio [HR] 0.34, 95% confidence interval 0.14–0.82, p = 0.0161). Particularly good survival was observed for patients with pCR in the durvalumab arm (n = 42 patients with no event).

To identify cellular pathways relevant for pCR and long-term outcome, we performed a gene set enrichment analysis (GSEA) using the 50 hallmark (HM) gene sets[19] as well as the 25 biological groups defined for the HTG Oncology Biomarker Panel (HTG gene sets). Up- or downregulation was quantified using the normalized enrichment score (NES). Based on gene expression data for 2,549 genes in 148 pretherapeutic biopsies, we performed a GSEA for both clinical endpoints, DDFS and pCR combining both therapy arms (Figure 2A), including HTG gene sets (HTG) and HM gene sets. The NES for pCR (x axis, blue) and DDFS (y axis, orange) is shown. In the upper right quadrant, pathways are enriched that are linked to increased pCR rate as well as improved DDFS, including several immune gene sets. In the lower right quadrant, pathways are enriched

CellPress
OPEN ACCESS

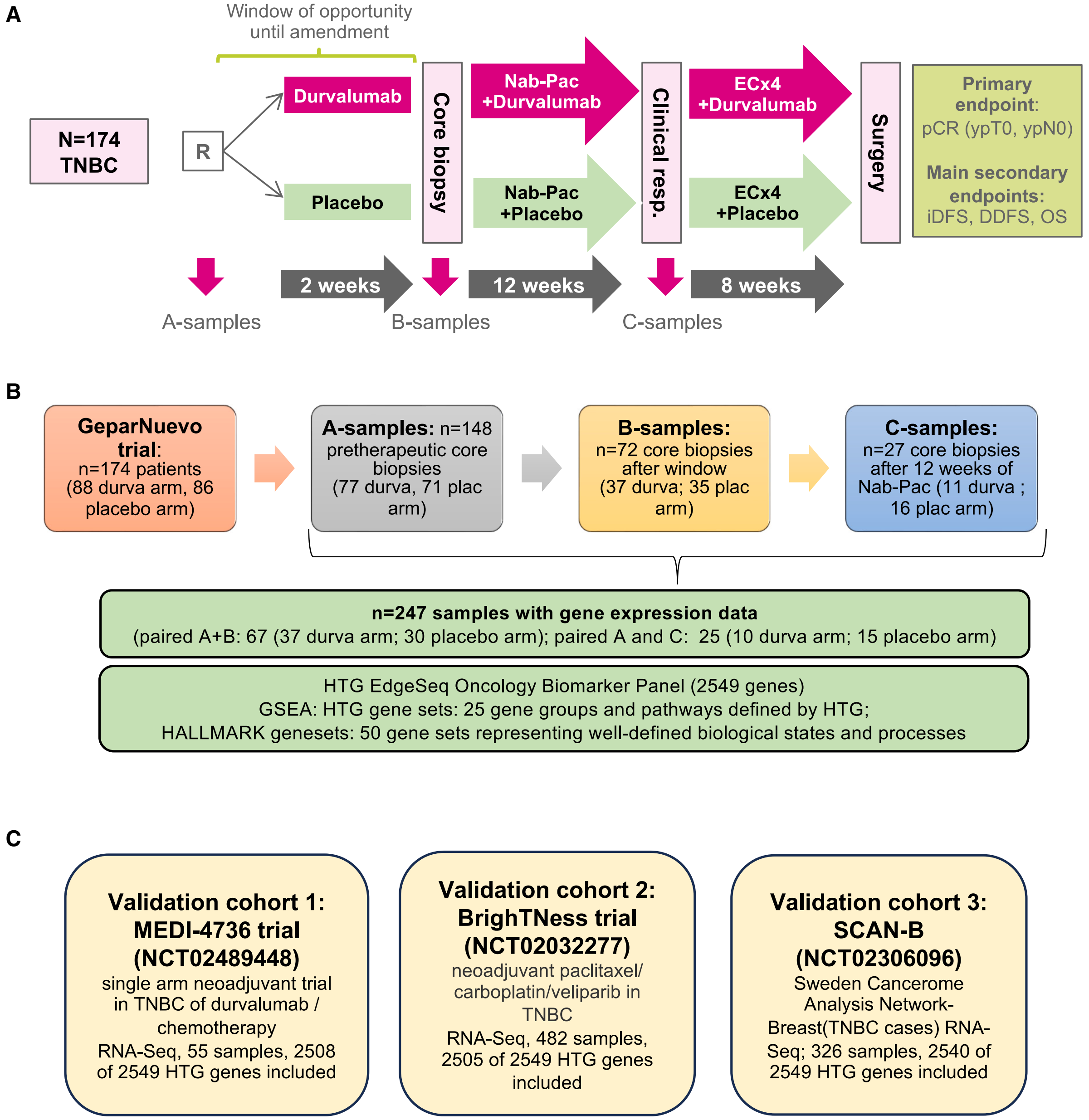

Figure 1. Clinical trial design and samples for biomarker analysis

(A) Overview of the neoadjuvant phase 2 double-blind randomized placebo-controlled GeparNuevo trial (NCT02685059).

(B) Longitudinal gene expression analysis for the evaluation of durvalumab and chemotherapy-related changes. Sequential formalin-fixed paraffin-embedded (FFPE) samples for longitudinal analysis: before the start of therapy (A-samples), after the window phase (B-samples), and after 12 weeks of chemotherapy +/− durvalumab (C-samples).

(C) Description of three gene expression cohorts for independent validation: MEDI-4736 trial (NCT02489448), BrighTNess trial (NCT02032277), SCAN-B (NCT02306096). iDFS, invasive disease-free survival; DDFS, distant disease-free survival; OS, overall survival; durva, durvalumab; plac, placebo; TNBC, triple-negative breast cancer; TILs, tumor-infiltrating lymphocytes; pCR, pathological complete response; GSEA, gene set enrichment analysis.

that are linked to increased pCR, but reduced survival, including proliferation pathways. In the lower left quadrant, several stromal gene sets are enriched. These gene sets have a negative correlation with both, pCR and DDFS, indicating a highly aggressive phenotype that does not seem to benefit from neoadjuvant chemotherapy. Figures S1–S4 show the details of the gene sets relevant for pCR and DDFS in the complete GeparNuevo cohort. Proliferation-related gene sets were positively associated with increased pCR, but not with improved DDFS. In contrast, an enrichment of immune-related gene sets was positively associated with both, increased pCR and improved DDFS.

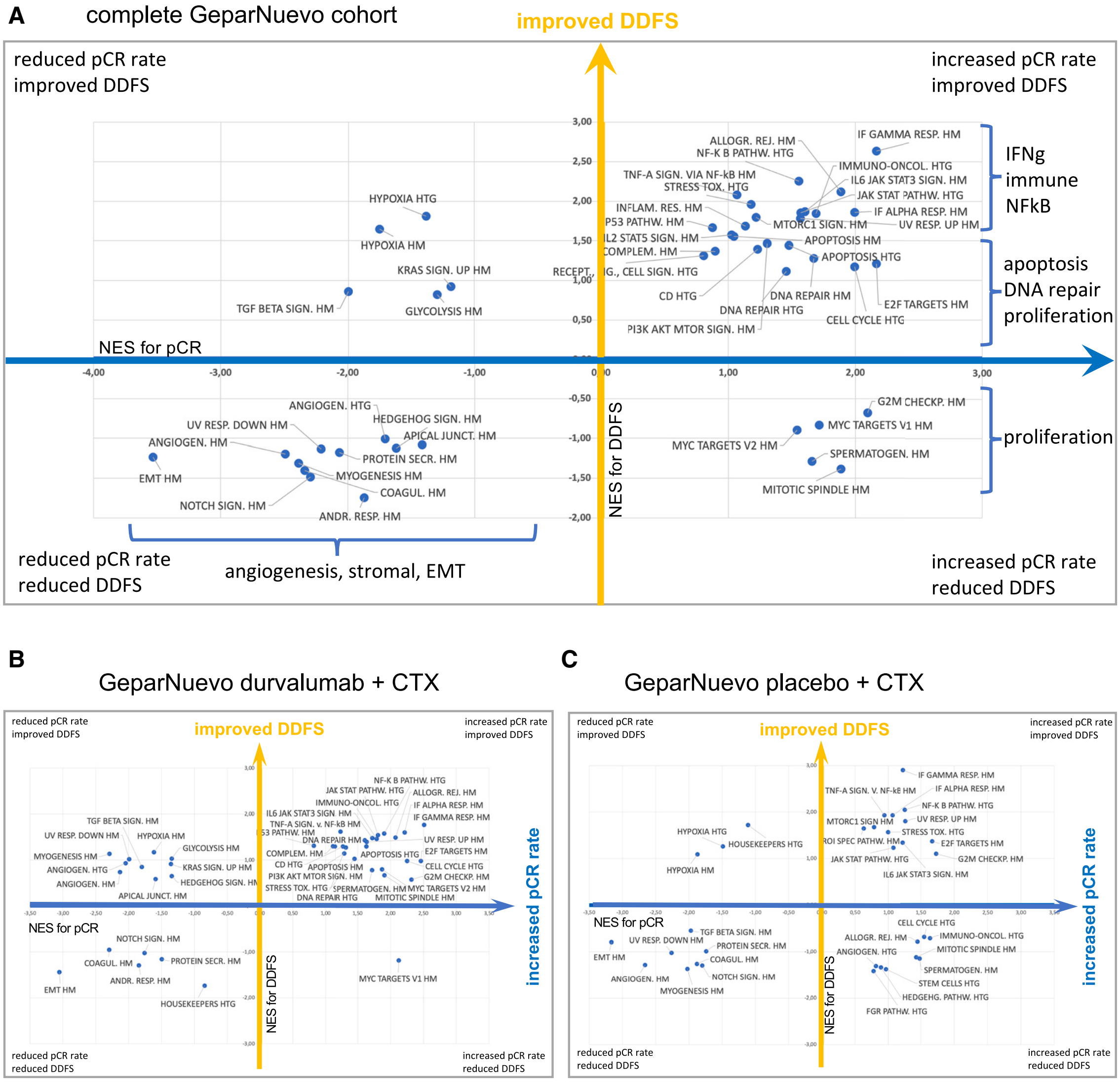

Figure 2. Gene set enrichment for the clinical endpoints pCR and DDFS

(A) 2D GSEA plot of the complete G9 biomarker cohort (both arms combined, pretherapeutic biopsies) using HTG-defined (HTG) and hallmark gene sets (HM). The normalized enrichment score (NES) for pCR (x axis, blue) and DDFS (y axis, orange) is shown.

(B) 2D GSEA plot for the durvalumab arm and the two clinical endpoints pCR and DDFS.

(C) 2D GSEA plot for the placebo arm and the two clinical endpoints pCR and DDFS.

Gene signatures relevant for pCR and/or DDFS—Stratified by treatment arms

We stratified the GSEA for both therapy arms (Figures 2B and 2C) and observed considerable differences of gene sets relevant for pCR and/or DDFS in the two therapy arms. In the 2D-GSEA plot for the durvalumab arm, the majority of cellular pathways are found in the upper right quadrant of the plot, showing gene sets linked to improved pCR rate as well as improved DDFS (Figure 2B). These gene sets include immune-related, but also proliferation gene sets. In contrast, gene sets linked to EMT and stromal parameters are found in the lower left quadrant, indicating a negative predictive role for pCR as well as a negative predictive role for DDFS.

In the placebo arm, the accumulation of gene sets in the upper right quadrant is not observed. In contrast, gene sets related to cellular proliferation are linked to increased pCR, but reduced DDFS (lower right quadrant, Figure 2C). Gene sets related to stromal biology have a similar negative role for pCR and DDFS as shown in the durvalumab arm.

A more detailed analysis of GSEA is shown in Figures S5 and S6 for DDFS and therapy response, with a focus on GSEA plots for the most important cellular pathways in the durvalumab arm (Figure S5) and the placebo arm (Figure S6).

In summary, the GSEA analysis shows an improved pCR and improved DDFS for immune, but also for proliferation-related gene sets in the durvalumab arm. Thus, the typical negative

CellPress
OPEN ACCESS

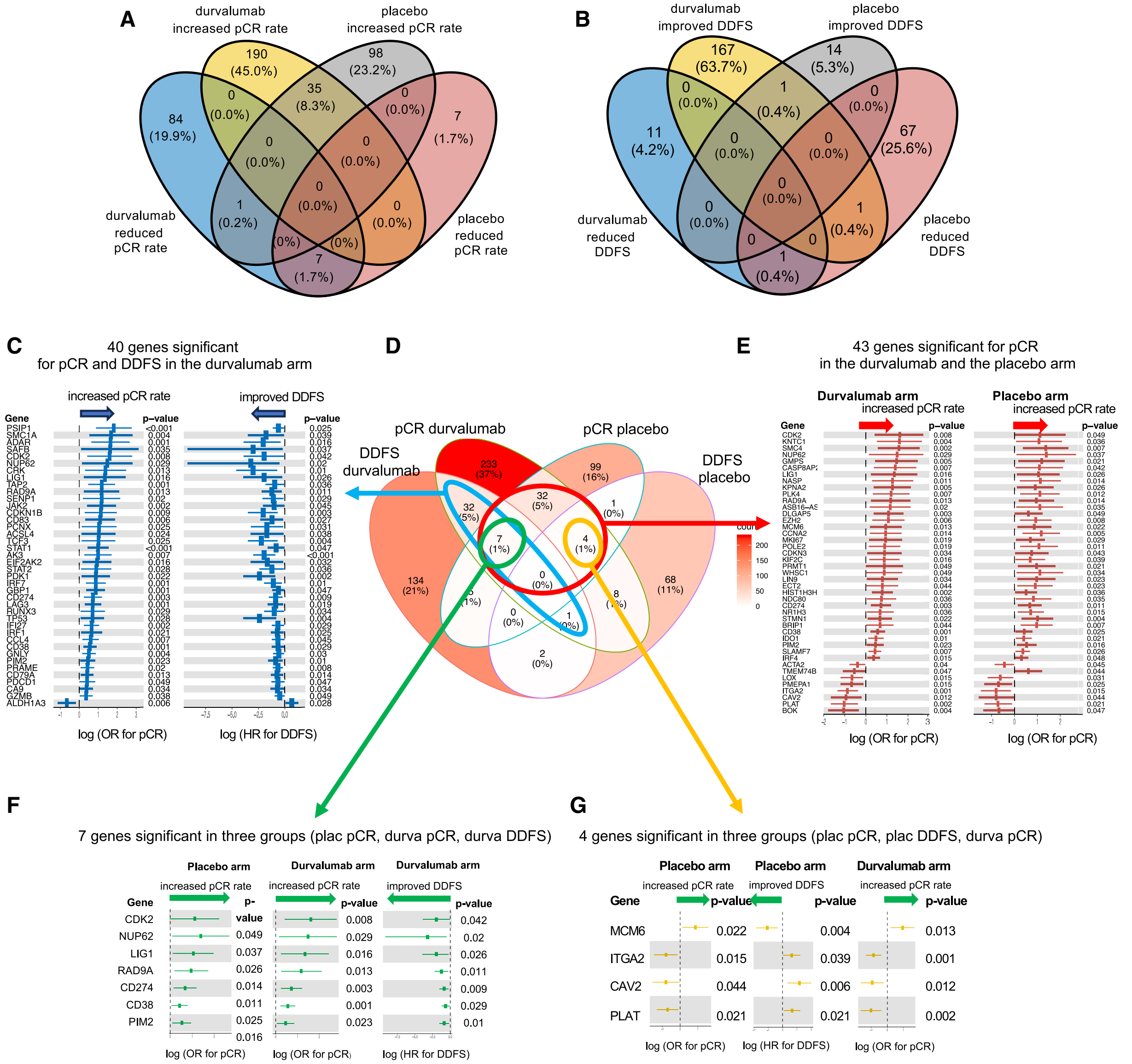

Figure 3. Relevance of individual genes for survival and therapy response in the two therapy arms

(A) Overview on the number of significant genes ($p < 0.05$) for increased or reduced pCR rate in the durvalumab arm as well as the placebo arm (Venn diagram).
(B) Overview on the number of significant genes ($p < 0.05$) for improved or reduced DDFS in the durvalumab arm as well as the placebo arm (Venn diagram).
(C–G) Overlap between significant genes for DDFS and pCR in the two trial arms, Venn diagram (D) as well as odds ratio (OR) for pCR and HR for DDFS for selected genes that are significant in more than one subgroup. Error bar: 95% CI.

role of proliferation markers for DDFS is not observed in the durvalumab arm, while it is still present in the placebo arm.

Prognostic and predictive genes in the two therapy arms

For a detailed analysis, we focused on the individual significant genes relevant for pCR and DDFS in the two therapy arms (Figure 3). In the durvalumab arm, a total of 317 genes were significantly correlated with pCR and a total of 181 genes were significantly correlated with DDFS ($p < 0.05$, Figures 3A and 3B). In the placebo arm, the number of significant genes was considerably lower: only 148 genes were significant for pCR and only 84 genes for DDFS (Figures 3A and 3B).

Interestingly, the overlap of genes in the different signatures (Figures 3C–3G) was very limited. Only 7 genes were significant for durvalumab pCR, placebo pCR, and durvalumab DDFS (Figure 3F), and 4 genes were significant for durvalumab pCR, placebo pCR, and placebo DDFS (Figure 3G). In the durvalumab arm, 40 genes were significant for both endpoints (pCR and DDFS, Figure 3C), while in the placebo arm, only 5 genes were significant for both endpoints (Figure 3D). A total of 43 genes were significant for pCR in the durvalumab as well as the placebo arm; most of these genes were immune-related genes (Figure 3E).

Based on the results of the GSEA, we defined three major gene set groups (GSGs) that contribute to outcome and prognosis in

the neoadjuvant setting. The immune GSG was defined as those genes included in the gene sets immuno-oncology (HTG), allograft rejection (HM), interferon alpha response (HM), and interferon-gamma response (HM). The proliferation-related GSG was defined as those genes included in the gene sets cell cycle (HTG), E2F targets (HM), G2M checkpoint (HM), and mitotic spindle (HM). The stromal-related GSG was defined as those genes included in the gene sets angiogenesis (HTG), angiogenesis (HM), coagulation (HM), epithelial-mesenchymal transition (HM), fatty acid metabolism (HM), and myogenesis (HM).

In Figure 4, the relevant genes from the immune, proliferation, and the stromal GSG for pCR in the durvalumab arm are shown. Most immune genes and most proliferation-related genes were linked to an increased pCR rate (Figures 4A and 4B). In contrast, the most significant genes within stromal GSG were linked to a reduced pCR rate (Figure 4B); this group contained genes related to cancer-associated fibroblasts (CAFs), as well as angiogenesis.

The results of the three major GSGs for the endpoint DDFS in the durvalumab arm are shown in Figures 4C and 4D. Immune genes were associated with improved prognosis; the most significant gene was CD274 (PD-L1, $p = 0.009$). Interestingly, proliferation-related genes were associated with improved prognosis in the durvalumab arm, validating the results observed for proliferation-related gene sets in the GSEA. The significant stromal genes for the DDFS endpoint in the durvalumab arm included predominantly immune-related genes, which were associated with improved survival.

Effects of durvalumab in the window phase

In the window phase of GeparNuevo, patients were treated with neoadjuvant durvalumab vs. placebo for 2 weeks, without the addition of chemotherapy. This allowed assessing durvalumab-induced gene expression changes by comparison of biopsies before (A-samples) and after the window treatment (B-samples). An overview of longitudinal gene expression changes is shown in Figure 5 for differentially expressed genes in the window phase in the durvalumab (Figure 5A) and the placebo arm (Figure 5B). Durvalumab induced an upregulation of immune genes (including LAG3, FKBP5, PD-L1 (CD274), and CXCL12) and stromal markers such as procollagen C endopeptidase enhancer 1 (PCOLCE) (Figure 5A). In the placebo arm, only limited alterations were observed in the window phase (Figure 5B).

In GSEA using the HM gene sets as well as the HTG gene sets, we observed a durvalumab-induced upregulation of immune gene sets, including immuno-oncology (HTG), interferon-gamma response (HM), and allograft rejection (HM) (Figures 5C and 5D) in the window phase. This induction was not observed in patients treated with placebo (Figures 5C and 5E).

In contrast, proliferation-related gene sets, in particular the gene sets cell cycle, G2M checkpoints, and E2F targets, were reduced in core biopsies treated with durvalumab (Figures 5C and 5D), while they were enriched in post-window core biopsies from the placebo arm (Figures 5C and 5E).

Therapy-induced alterations after 12 weeks of chemotherapy with or without durvalumab

Additional biopsies (C-samples) were collected after 12 weeks of nab-paclitaxel therapy to investigate gene expression patterns with a durvalumab-chemotherapy combination compared to chemotherapy-placebo. The administration of chemotherapy in both treatment arms induced extensive changes in gene set enrichment profiles, with a total of 593 genes significantly altered by durvalumab-chemotherapy and a total of 606 genes by chemotherapy alone (data not shown).

GSEA after four cycles of chemotherapy shows a downregulation of the large majority of gene sets (Figure S7). However, within the durvalumab-chemotherapy arm, there were two immune-related gene sets that remained significantly increased (CD antigens [$p < 0.0001$] and immuno-oncology [$p < 0.0001$]). These increased immune gene sets were not observed in the placebo-chemotherapy arm. Similarly, the significant reduction of proliferation-related gene sets such as G2M checkpoint ($p < 0.0001$), EF2 targets ($p < 0.0001$), and cell cycle ($p < 0.0001$) by durvalumab was also similar between the window phase and the first four cycles of chemotherapy (Figures S7A and S7B). In the placebo-chemotherapy arm, the reduction of proliferation-related gene sets was observed only as a non-significant trend (Figures S7A and S7C). For several other gene sets, including hypoxia, MTORC signaling, glycolysis, and others, there was a significant reduction only in the durvalumab-chemotherapy combination arm. A significant reduction in both therapy arms is observed for several other gene sets (Figure S7).

In summary, durvalumab induced an activation of immune gene expression as well as a reduction of tumor proliferation, indicating a combination of immune activation with anti-proliferative tumor effects. Interestingly, these alterations were already observed after one dose of durvalumab in the window phase and persisted during the neoadjuvant chemotherapy phase.

Combination of gene signatures for pCR and survival

An overview of the results of the GeparNuevo investigation with a focus on defined GSGs is given in Figure 6. Individual genes from the 3 main GSGs (immune, proliferation, and stroma) are shown to illustrate important longitudinal alterations as well as differences between genes and pathways for pCR and DDFS in the two trial arms.

In the scatterplot in Figures 6A and 6B, genes with a positive impact on pCR and DDFS are shown in the upper right quadrant, including immune genes and proliferation-related genes, particularly in the durvalumab arm (Figure 6A). Genes with a negative impact on pCR as well as DDFS are located in the lower left quadrant, including stromal genes. The volcano plot in Figure 6C summarizes the regulation of immune, proliferation, and stromal genes by one dose of durvalumab in the window phase. Figure 6 illustrates that immune genes linked to improved outcome are induced in the durvalumab window, while proliferation genes are reduced in the window. The activation of immune pathways in the window phase may contribute to a coordinated anti-tumor response that is manifested by an induction of immune genes as well as a reduction of proliferation genes.

Interestingly, an induction of stromal genes was also observed in the durvalumab window. These stromal genes play a special

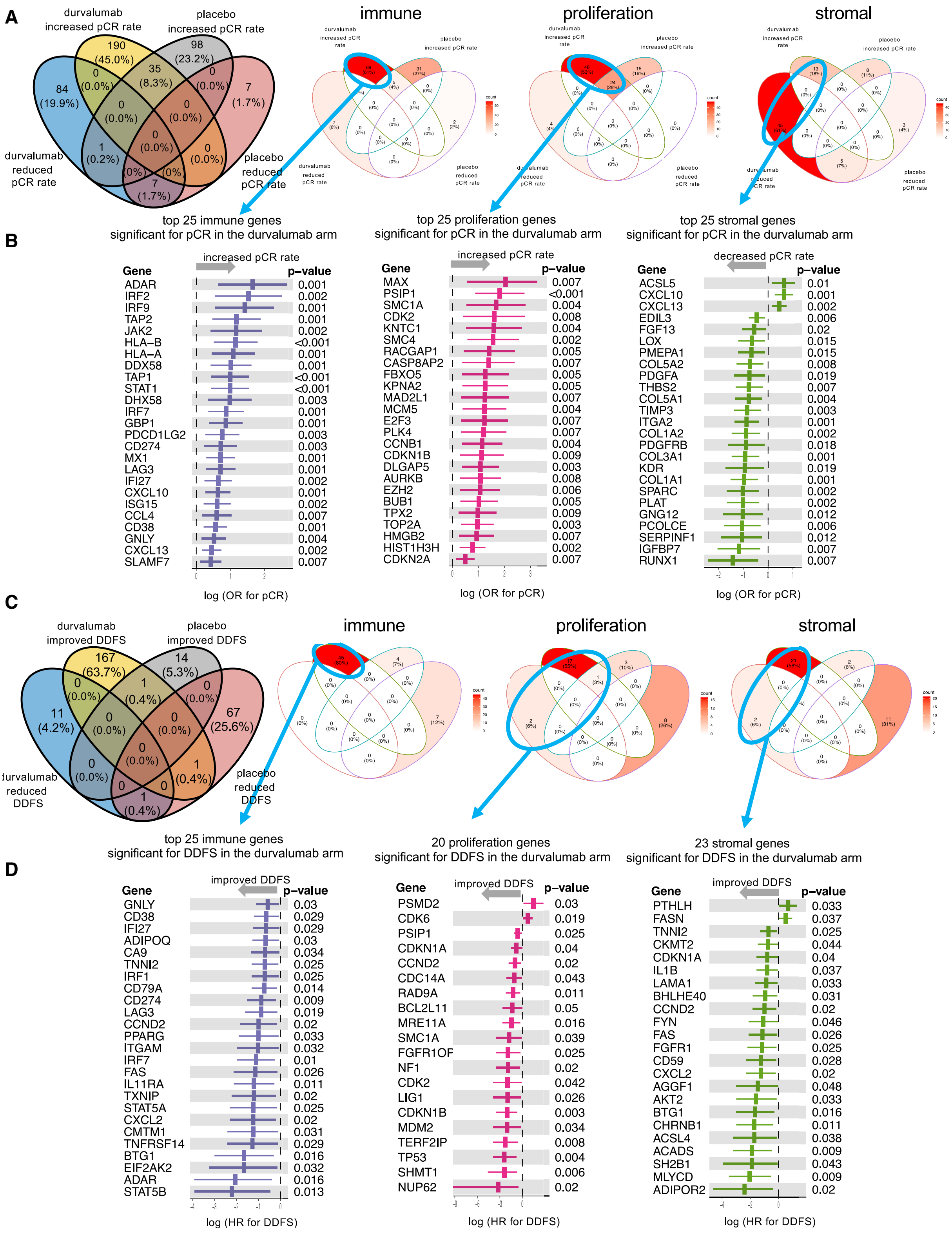

Figure 4. Contribution of the combined immune, proliferation, and stromal gene set groups to prediction of pCR

(A and B) Venn diagrams (A) as well as OR for pCR for selected genes from the three groups (B).

(C and D) Contribution of combined immune, proliferation, and stromal gene set groups (GSGs) to DDFS Venn diagrams (C) as well as HR for DDFS for selected genes from the three groups (D). Error bar: 95% CI.

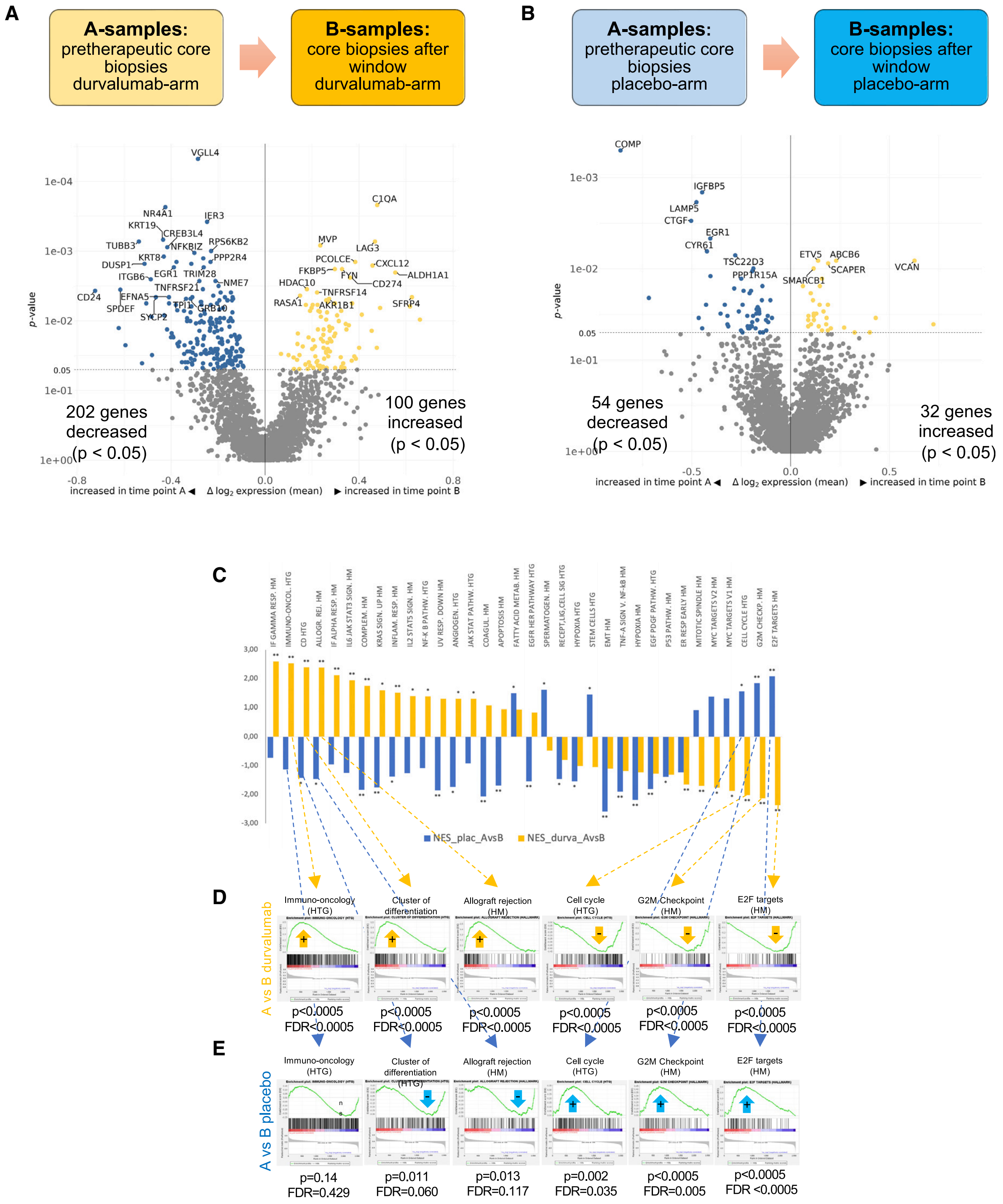

Figure 5. Evaluation of molecular alterations in longitudinal TNBC samples, comparing pretherapeutic core biopsies (A-samples) with core biopsies after the window phase (B-samples)

(A and B) Volcano plots for the evaluation of differentially expressed genes in the window phase in the durvalumab (A) and the placebo arm (B).

(C) Comparison of gene set enrichment in both therapy arms in the window phase of the trial. The normalized enrichment score (NES) for placebo (blue) and durvalumab (orange) is shown.

(D and E) GSEA results for selected gene sets in the window phase with (D) or without (E) durvalumab. CTX, chemotherapy; FDR, false discovery rate.

CellPress
OPEN ACCESS

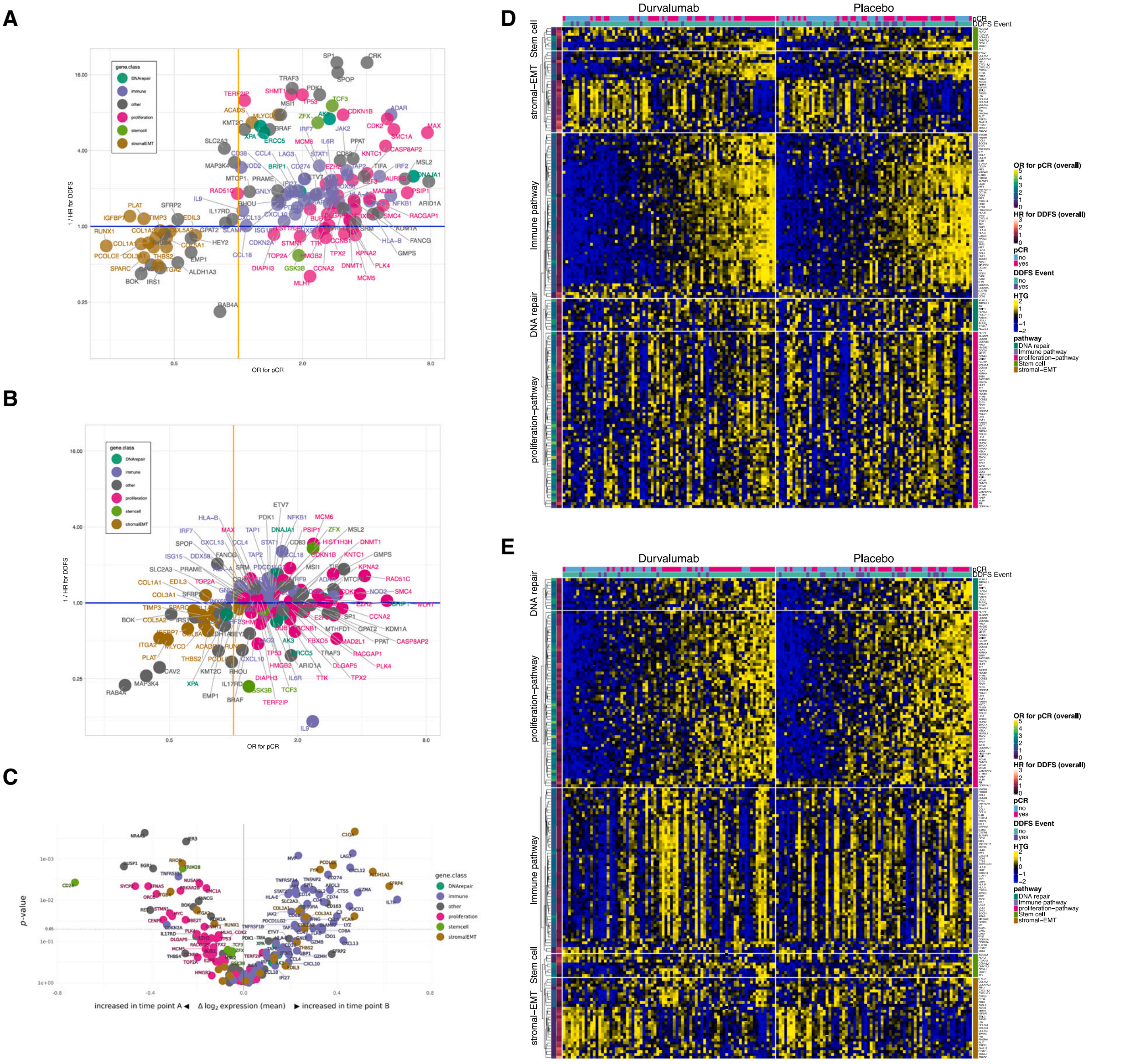

Figure 6. Longitudinal gene expression changes and predictive and prognostic genes in GeparNuevo—Summary of main results for individual genes from the gene groups

(A and B) Significant genes for pCR (OR, x axis) and DDFS (1/HR, y axis) for the durvalumab (A) and the placebo (B) arm of the GeparNuevo trial. Genes were selected based on significance ($p < 0.01$) for at least one endpoint (pCR and DDFS) in at least one of the study arms. Color coding: combined immune, proliferation, stromal gene groups; for genes that were linked to more than one group, immune and proliferation were the preferred groups.

(C) Volcano plot highlighting longitudinal alterations induced by durvalumab during the window phase for selected genes relevant for immune activation, proliferation, and stromal remodeling.

(D and E) Overview of gene expression data from all pretherapeutic biopsies, separated by trial arm (left, durvalumab arm, right: placebo arm). Genes were selected based on a significance ($p < 0.01$) for at least one endpoint (pCR or DDFS) in at least one trial arm or in the complete cohort. Genes were sorted into the combined immune, proliferation, and stromal gene group (as indicated in the STAR Methods section). In addition, the gene groups DNA repair and stem cell were included. Genes that were assigned into more than one gene group were duplicated for this figure. The heatmap in (D) is sorted based on the combined immune gene set; the heatmap in (E) is sorted based on the combined proliferation gene set.

role: they are induced by durvalumab, similar to the immune genes. But—in contrast to the immune genes—they are linked to poor outcome with reduced pCR rate and reduced survival.

Figures 6D and 6E summarize the alterations of the main GSGs in the durvalumab and the placebo arm, sorted by increased expression of immune genes (Figure 6D) or increased expression of proliferation genes (Figure 6E). In both figures, the stromal genes are inversely related to the immune or proliferation genes, with increased expression of stromal genes in non-responding patients.

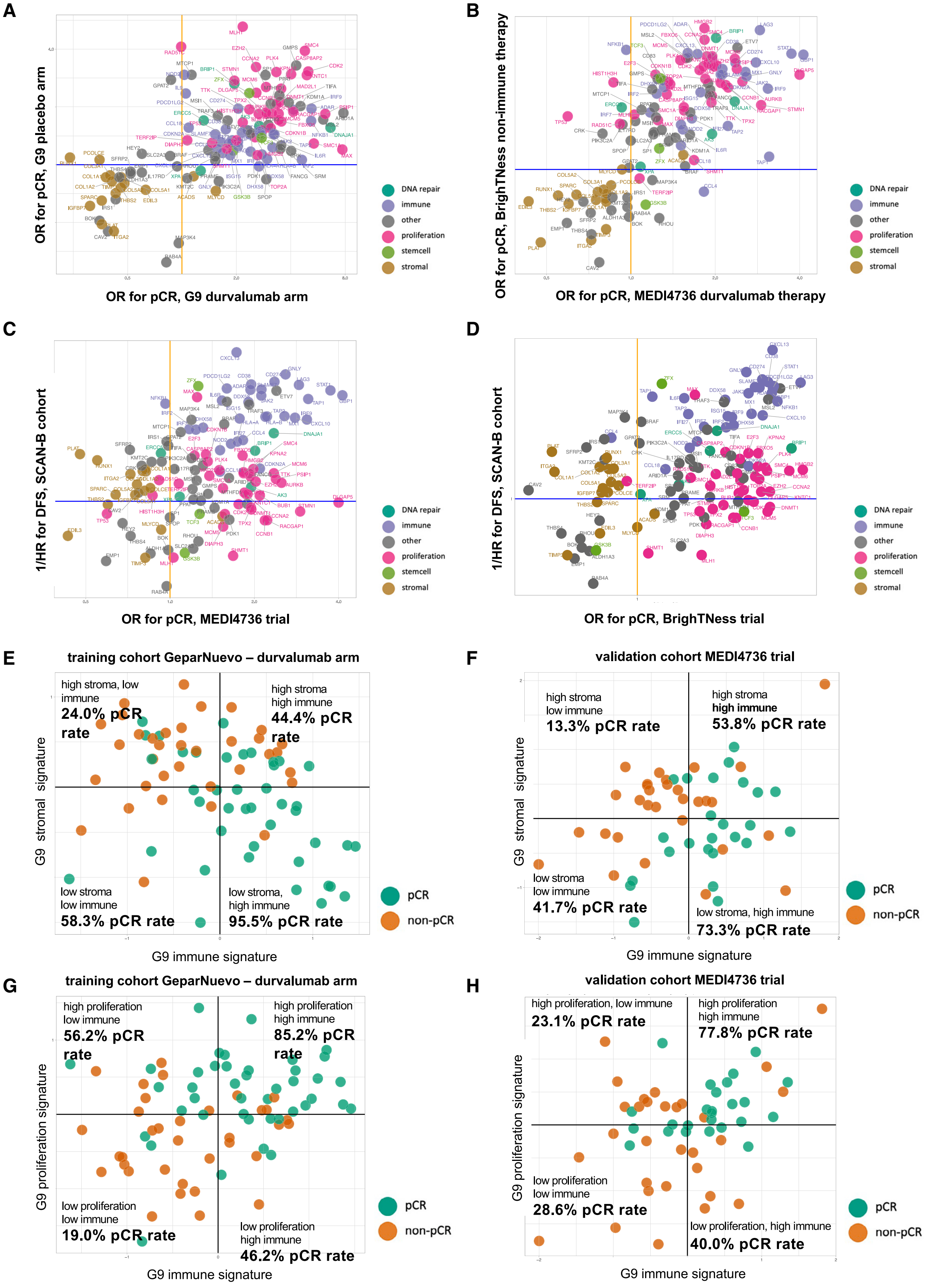

(legend on next page)

Validation in independent datasets

For an independent validation of the associations of the different gene groups with clinical endpoints and treatment arms, we used the three validation cohorts MEDI4736 (neoadjuvant durvalumab therapy), BrighTNess (neoadjuvant non-durvalumab therapy), and SCAN-B (survival data with non-immune therapy). As shown in Figures 7A and 7B, the role of stromal, proliferation, and immune genes for pCR was very similar when comparing the two treatment arms of GeparNuevo (Figure 7A) and the combined MEDI4736 and BrighTNess dataset (Figure 7B), validating the positive role of immune and proliferation genes as well as the negative role of stromal genes for therapy response. In addition, regarding the combined pCR and DDFS results (as shown in Figures 6A and 6B for the two GeparNuevo arms), a similar distribution of the main gene groups (stromal, immune, and proliferation) was observed in a combination of MEDI4736 (Figure 7C) or BrighTNess (Figure 7D) with the SCAN-B cohort to cover the survival endpoint.

Based on genes from stromal, immune, and proliferation GSGs, which were predictive for pCR in the durvalumab arm of the GeparNuevo (G9) training cohort, we generated three gene signatures (see supplemental methods), which were predictive for pCR in GeparNuevo (Figures 7E and 7G). We validated the predictive value of these three signatures in the MEDI4736 validation cohort (Figures 7F and 7H). The combination of stromal and immune signatures (Figures 7E and 7F) as well as proliferation and immune signatures (Figures 7G and 7H) defined patient groups with different pCR rates in both cohorts. In particular, tumors with high stromal and low immune gene expression had a low pCR rate of 24.0% in the GeparNuevo durvalumab arm (Figure 7E) and 13.3% in the MEDI4736 cohort (Figure 7F), and tumors with low stromal and high immune gene expression had a high pCR rate of 95.5% in the GeparNuevo durvalumab arm (Figure 7E) and 73.3% in the MEDI4736 cohort (Figure 7F). Tumors with high immune gene expression as well as high proliferation-related gene expression had a pCR rate of 85.2% in GeparNuevo (Figure 7G) and 77.8% in MEDI4736 (Figure 7H).

Figure S8 shows the distribution of patients with pCR depending on the three gene signatures covering immune, proliferation, and stromal gene expression in the GeparNuevo durvalumab arm (training cohort) and the validation cohort MEDI4736. In particular, the negative role of stromal gene expression as well as the positive role of immune and proliferation genes could be validated in the independent MEDI4736 cohort.

DISCUSSION

In this study using serial biopsies from the neoadjuvant GeparNuevo trial, we characterized the longitudinal molecular changes induced by neoadjuvant immunotherapy and chemotherapy and their association with response, survival, and immunotherapy resistance. The main results of this investigation were: (1) administration of one dose of durvalumab alone increased immune gene expression and reduces proliferation-related gene expression; (2) these durvalumab-induced effects persist in the chemotherapy phase; (3) for chemotherapy alone, we could not identify a coordinated cellular response, just a downregulation of all major cellular pathways; (4) the addition of durvalumab to chemotherapy leads to a change in predictive and prognostic gene expression patterns, with a positive effect of immune genes but also a positive effect of proliferation genes for pCR as well as DDFS (which is in line with the reduction of proliferation observed already in the window phase); and (5) we identify stromal-related gene expression as a contributor to immunotherapy resistance and poor prognosis.

In our study, we show that immune biomarkers are linked to therapy response as well as improved survival, suggesting that the modulation of tumor-immune interaction could be an option to improve outcome. Considering the significant reduction of proliferation-related gene expression that was already observed in the durvalumab window, the observed altered prognostic effect of tumor proliferation might be caused by durvalumab-induced immune activation, which might lead to an inhibition of proliferation pathways by the activated immune system. Therefore, the effect of durvalumab is already emerging within the window phase, where a single dose of durvalumab induces exactly those changes that are later relevant for prognosis, i.e., an increased immune gene activation and a reduction of proliferation-related pathway activity.

This suggests that the alterations by durvalumab are observed early during treatment, suggesting a role for immunotherapy particularly in the neoadjuvant setting. This is in line with the result of recent trials in melanoma,[20,21] and also with emerging preclinical and clinical evidence in breast cancer.[22,23] Considering this early immune induction by immunotherapy, the relevance of the post-neoadjuvant continuation of immunotherapy should be evaluated in additional clinical trials, in particular in patients with pCR.

PD-L1 (CD274) expression was significantly increased in patients obtaining a pCR in the durvalumab as well as the placebo arm, which is in line with previous observations, showing that PD-L1 is not a specific predictor of immunotherapy response, but a general marker of response to neoadjuvant therapy.[7,21,24] Interestingly, the most significant prognostic factor for improved DDFS in the durvalumab arm was the therapy target PD-L1 (CD274) itself. This prognostic effect of PD-L1 for improved DDFS was observed only in the durvalumab and not in the placebo arm.

Other immune checkpoint markers including PDCD1 (PD-1), PDCDLG2 (PD-L2), and LAG3 were also upregulated during the window phase, i.e., with one single dose of durvalumab. Combinations of immunotherapeutics have been evaluated in

Figure 7. Validation in three independent cohorts

MEDI4736 (neoadjuvant durvalumab therapy), BrighTNess (neoadjuvant non-durvalumab therapy), and SCAN-B (survival data with non-immune therapy).

(A and B) Stromal, proliferation, and immune genes for pCR in the two treatment arms of the GeparNuevo dataset (A) and the combined MEDI4736 and BrighTNess dataset (B). Similar distribution of the three main gene groups (stromal, immune, and proliferation) if the MEDI4736 (C) or BrighTNess (D) were combined with the SCAN-B cohort to cover the survival endpoint. In the GeparNuevo durvalumab arm as well as the MEDI4736 cohort, the combination of stromal and immune gene signatures (E, F) as well as proliferation and immune signatures (G, H) defined patient groups with similar pCR rates in both cohorts.

malignant melanoma[25,26] and other malignancies,[27,28] but their role in early breast cancer is still not clear.

Despite this upregulation of immunosuppressive molecules, the upregulation of indicators of cytotoxic activities such as granzyme A and interferon gamma, the upregulation of T cell markers including CD8A and CD4, as well as the upregulation of elements of the antigen-presenting machinery, such as HLA-E and TAP1, indicate an increased cytotoxic activity already within the durvalumab window. This cytotoxic activity is in line with the reduction of tumor proliferation-related gene expression that is also observed during the window phase and might contribute to positive association of higher initial tumor proliferation with improved DDFS, which is observed only in the durvalumab arm.

In addition to the effects on immune activation, the modulation of the stromal microenvironment by durvalumab includes also an induction of stromal gene expression, which has a negative impact on pCR. This durvalumab-induced stromal response might suggest an emerging resistance mechanism, which might be related to a modulation of the tumor microenvironment. The genes COL1A1, COL1A2, and COL3A1, as well as the PCOLCE enzyme were significantly induced by durvalumab and were associated with a reduced pCR rate in the durvalumab arm. PCOLCE is involved in the enzymatic cleavage of procollagen to collagen[29] and has been described as a negative prognostic marker in gastric cancer.[30] In addition, it has been shown that PCOLCE is relevant for lumen formation in angiogenesis.[31] Considering the fact that stromal fibroblasts are the main sources of collagens, our results suggest an induction of CAF activity by immune checkpoint inhibitors, which contributes to therapy resistance and modulates angiogenesis.

In addition, an important step in the generation of an immunosuppressive tumor microenvironment is the increased expression of the chemokine CXCL12, which has also been observed in the durvalumab window. CXCL12 is produced by CAFs and has been shown to induce angiogenesis[32] and to induce the differentiation of monocytes into immunosuppressive tumor-associated macrophages (TAMs).[33] In line with this finding, we observed an increased expression of the macrophage marker CD68 in the durvalumab window.

For an independent validation, we combined two separate trial cohorts (MEDI4736 and BrighTNess). In this combination, we could validate the negative role of stromal gene expression on pCR. However, it should be noted that the non-randomized combination of two trial cohorts is a potential limitation of our study. Based on the results of our study as well as studies in other tumor types, concepts for combination therapies targeting the angiogenesis, tumor microenvironment, and CAFs in parallel to immunotherapy are emerging.

In our study, we describe the induction of genes relevant for CAF and TAM activity by a PD-L1 inhibitor in a neoadjuvant breast cancer clinical trial cohort. Our results support the role of these cell types for therapy resistance in other types of tumors[34,35] and for tumor progression and generation of an immune-suppressive microenvironment in breast cancer.[36–39] Based on our results, a model of adaptation of the microenvironment to immune checkpoint inhibitor therapy is emerging that leads to a better understanding of the mechanisms of therapy response and resistance.

The identification of stromal cells as an important contributor to immunotherapy resistance provides translational evidence for new therapeutic options to overcome this resistance. We have specifically focused on the expression of genes relevant for fibroblast activation and angiogenesis that could be targeted by angiogenesis or FGFR inhibitors as well as multi-kinase inhibitors.[40,41] In particular, bispecific antibodies targeting the immune system as well as angiogenesis[42–44] might provide a promising option to modulate the tumor microenvironment in TNBC and other tumor types.

The results from our investigation strongly support the further development of these therapy approaches in TNBC and contribute to the current research focus on stromal alterations and modulation of the tumor microenvironment across tumor entities.

Limitations of the study

It should be noticed that there are several limitations of this study: we have not performed formal correction for multiple testing of *p* values of gene expression experiments. Furthermore, it is not possible at this stage to precisely identify the cell type (e.g., CAF or macrophage) that is responsible for the control of the stromal modeling. Additional validations in prospective clinical trial cohorts are necessary for the validation of the combined gene expression signature.

RESOURCE AVAILABILITY

Lead contact

Further information and requests for resources and reagents should be directed to and will be fulfilled by the lead contact, Sibylle Loibl (sibylle.loibl@gbg.de).

Materials availability

This study did not generate new unique reagents.

Data and code availability

- The data for this study, together with analysis code and output files, are available on GitHub (Link: https://github.com/tkarn/G9-HTG).
- The code for this analysis has been deposited in Zenodo (link: https://doi.org/10.5281/zenodo.13833943).
- Any additional information required to reanalyze the data reported in this work paper is available from the lead contact upon request.

ACKNOWLEDGMENTS

The analysis was funded by a research grant from the German Cancer Aid (Deutsche Krebshilfe, Translational Oncology, Integrate-TN project; 70113450). The validation analysis was funded by a research grant from the German Federal Ministry of Science and Education (BMBF, SATURN3 project; 01KD2206M) and by grants from the H.W. & J. Hector Stiftung (M2327) and from ERA-NET TRANSCAN (MAGNOLIA). Research and biostatistical resources were provided by GBG.

AUTHOR CONTRIBUTIONS

The study was designed by C.D., A.S., J.R., T.K., A.H., K.E.W., S.R., V.N., and S.L. C.D., A.S., J.R., T.K., A.H., M.B., J.H., P.J., H.-P.S., D.-M.Z., B.F., C.H., J.T.-S., F.M., T.F., J.T., B.V.S., T.S., M.M., J.-U.B., M.v.M., C. Schem, P.S., T.L., V.M., E.S., D.G.S., C. Solbach, O.M.-F., C.J., C.E.G., P.A.F., L.P., V.N.,

M.U., and S.L. contributed to data acquisition. Data analysis was performed by C.D., J.R., T.K., A.H., K.E.W., S.R., P.J., J.T.-S., B.V.S., T.S., M.M., D.G.S., O.M.-F., C.J., C.E.G., L.P., V.N., and S.L. Patient recruitment, sample collection, as well as data collection were performed by C.D., A.S., J.R., T.K., A.H., K.E.W., S.R., M.B., J.H., P.J., H.-P.S., D.-M.Z., B.F., C.H., J.T.-S., F.M., T.F., J.T., B.V.S., T.S., M.M., J.-U.B., M.v.M., C. Schem, P.S., T.L., V.M., E.S., D.G.S., C. Solbach, O.M.-F., C.J., C.E.G., P.A.F., L.P., V.N., M.U., and S.L. All authors interpreted the data. The first draft of the report was written by C.D. Verification of the underlying data was performed by C.D., J.R., T.K., and A.H. The decision to submit the report for publication was made by all the authors. All authors contributed to the review of the manuscript.

DECLARATION OF INTERESTS

C.D. reports grants from European Commission H2020, grants from German Cancer Aid Translational Oncology, grants from German Breast Group, and grants from BMBF to the institution during the conduct of the study; personal fees from Novartis, personal fees from Roche, personal fees from MSD Oncology, personal fees from Daiichi Sankyo, personal fees from AstraZeneca and Molecular Health, grants from Myriad, personal fees from Merck, and other funding from Sividon Diagnostics outside the submitted work; in addition, C.D. has a patent VMScope digital pathology software with royalties paid, a patent WO2020109570A1—cancer immunotherapy pending, and a patent WO2015114146A1 and WO2010076322A1—therapy response issued. J.R. declares to be a GBG Forschungs GmbH employee. GBG Forschungs GmbH received funding for research grants from AbbVie, Amgen, AstraZeneca, BMS, Daiichi Sankyo, Gilead, Molecular Health, Novartis, Pfizer, and Roche (paid to the institution). Funding was also received (non-financial/medical writing) from Daiichi Sankyo, Gilead, Novartis, Pfizer, Roche, and Seagen (paid to the institution). GBG Forschungs GmbH has royalties in VM Scope and patents pending: EP14153692.0, EP21152186.9, and EP15702464.7. T.K. reports a patent WO2020109570A1 pending. S.R. declares to be a GBG Forschungs GmbH employee. GBG Forschungs GmbH received funding for research grants from AbbVie, Amgen, AstraZeneca, BMS, Daiichi Sankyo, Gilead, Molecular Health, Novartis, Pfizer, and Roche (paid to the institution). J.H. reports research funding from Lilly; honoraria from Lilly, Novartis, Roche, Pfizer, AstraZeneca, Seagen, Gilead, and Daiichi; consulting and advisory relationships with Lilly, Novartis, Roche, Pfizer, AstraZeneca, Gilead, and Daiichi; travel expenses from Roche, Novartis, Daiichi, and Gilead. P.J. reports research funding and travel expenses from Gilead Sciences GmbH. C.H. reports an advisory role and speakers bureau role for AstraZeneca, Roche, Novartis, and Aristo Pharma. B.V.S. is an employee of BioNTech SE and reports a patent WO2020109570A1 pending. J.-U.B. reports consultation fees, honoraria, and reimbursement for attending symposia from AstraZeneca, Amgen, Daiichi Sankyo, Eisai, Gilead, Lilly, MSD, Novartis, Pfizer, Roche, and Seagen. M.v.M. reports personal fees, honoraria, or travel grants from Amgen, AstraZeneca, Daiichi Sankyo, Genomic Health, GSK, Lilly, Molecular Health, MSD, Mylan, Novartis, Pfizer, Pierre Fabre, Roche, and Seagen.

C. Schem reports speaker activities for Roche, Pfizer, Novartis, Celgen, Novartis, Exact Sciences, MSD, AstraZeneca, Lilly, and Seagen, as well as advisory boards for Roche, Astra Zeneca, Pfizer, Novartis, MSD, Amgen, Exact Sciences, Stemline, Lilly, and Novartis.

T.L. reports personal fees from Amgen, Roche, Teva, Clovis, Tesaro, MSD, Novartis, Pfizer, Lilly, GSK, Gilead, AstraZeneca, Daiichi Sankyo, Stemline, and Seagen outside of the submitted work. T.L. participates in advisory boards from Amgen, MSD, Tesaro, Roche, Pfizer, Lilly, Myriad, Esai, GSK, Gilead, Daiichi Sankyo, Roche, and AstraZeneca outside of the submitted work and T.L. received travel support from Pfizer, PharmaMar, MSD, Celgene, Roche, AstraZeneca, Gilead, Daiichi Sankyo, Stemline, and Clovis outside of the submitted work. P.S. reports grants, personal fees, and non-financial support from AbbVie, Amgen, AstraZeneca, Bristol Myers Squibb Company, MSD, Incyte, Janssen-Cilag, Novartis, Takeda, Pfizer, and Roche. V.M. received speaker honoraria from AstraZeneca, Daiichi Sankyo, Eisai, GSK, Pfizer, MSD, Medac, Novartis, Roche, Seagen, Onkowissen, high5 Oncology, Medscape, Gilead, and Pierre Fabre; consultancy honoraria from Roche, Pierre Fabre, Amgen, ClinSol, Novartis, MSD, Daiichi Sankyo, Eisai, Lilly, Sanofi, Seagen, Gilead, and Stemline; institutional research support from Novartis, Roche, Seagen, and Genentech; and travel grants from Roche, Pfizer, Daiichi Sankyo, and Gilead. L.P. has received consulting fees and honoraria for advisory board participation from Pfizer, AstraZeneca, Merck, Novartis, Bristol Myers Squibb, GlaxoSmithKline, Genentech/Roche, Personalis, Daiichi, Natera, and Exact Sciences and institutional research funding from Seagen, GlaxoSmithKline, AstraZeneca, Merck, Pfizer, and Bristol Myers Squibb. C.E.G. reports the following competing interests: Exact Sciences, Advisory Board, personal; AbbVie, Steering Committee member, institutional, co-chair of SC for BrighTNess; Daiichi Sankyo, member SC, institutional, co-chair of SC for DESTINY-Breast05; Genentech/Roche, SC member, institutional, co-chair of SC for lidERA; Genentech/Roche, coordinating PI, institutional, NSABP B-59/GeparDouze; and Genentech/Roche, SC member, institutional, co-chair of SC for KATHERINE. C.J. reports honoraria from AstraZeneca, Amgen, Daiichi Sankyo, Lilly, Roche, Pfizer, MSD Oncology, Pierre Fabre, Sanofi-Aventis, Seagen, Gilead, and Novartis and has a consulting or advisory role for Amgen, Lilly, Roche, Pfizer, Pierre Fabre, Novartis, MSD Oncology, Agendia, Seagen, Gilead, Lilly, Stemline, and Medac. V.N. declares to be a GBG Forschungs GmbH employee. GBG Forschungs GmbH received funding for research grants from AbbVie, AstraZeneca, BMS, Daiichi Sankyo, Gilead, Novartis, Pfizer, and Roche (paid to the institution). GBG Forschungs GmbH received other funding from Daiichi Sankyo, Gilead, Novartis, Pfizer, Roche, and Seagen (paid to the institution). GBG Forschungs GmbH has the following royalties/patents: EP14153692.0, EP21152186.9, EP15702464.7, EP19808852.8, and VM Scope GmbH. M.U. reports honoraria from AstraZeneca, Amgen, Daiichi Sankyo, Lilly, Roche, Pfizer, MSD Oncology, Pierre Fabre, Sanofi-Aventis, Myriad, Seagen, Gilead, and Novartis; has a consulting or advisory role for Amgen, Lilly, Roche, Pfizer, Pierre Fabre, Novartis, MSD Oncology, Agendia, Seagen, Gilead, Lily, Stemline, Genzyme, and Medac; and all honoraria and fees are paid to the employer/institution. S.L. reports grants and other funding from AbbVie; other funding from Amgen; grants and other funding from AstraZeneca; other funding from BMS; grants and other funding from Celgene; grants, non-financial support, and other funding from Daiichi Sankyo; other funding from EirGenix; other funding from Eisai Europe Ltd; other funding from GSK; grants, non-financial support, and other funding from Immunomedics/Gilead; other funding from Lilly; other funding from Merck; grants from Molecular Health; grants, non-financial support, and other funding from Novartis; grants, non-financial support, and other funding from Pfizer; other funding from Pierre Fabre; other funding from Relay Therapeutics; grants, non-financial support, and other funding from Roche; other funding from Sanofi; non-financial support and other funding from Seagen; and other funding from Olema Pharmaceuticals, outside the submitted work. In addition, S.L. has a patent EP14153692.0 pending, a patent EP21152186.9 pending, a patent EP15702464.7 issued, a patent EP19808852.8 pending, and a patent Digital Ki67 Evaluator with royalties paid.

STAR★METHODS

Detailed methods are provided in the online version of this paper and include the following:

- KEY RESOURCES TABLE
- EXPERIMENTAL MODEL AND STUDY PARTICIPANT DETAILS
  - Study design and clinical cohorts
- METHOD DETAILS
  - Endpoints
  - Gene expression analysis using the HTG OBP panel
  - Analysis of cellular pathways based on temporal expression data
  - Gene set groups (GSG) and gene class assignment
  - Predictive gene signatures
- QUANTIFICATION AND STATISTICAL ANALYSIS
  - Statistical analysis
  - Correction for multiple testing
  - Organization of the bioinformatical analysis

SUPPLEMENTAL INFORMATION

Supplemental information can be found online at https://doi.org/10.1016/j.xcrm.2024.101825.

Received: August 6, 2023
Revised: June 3, 2024
Accepted: October 18, 2024
Published: November 19, 2024

REFERENCES

1. Carey, L.A., Dees, E.C., Sawyer, L., Gatti, L., Moore, D.T., Collichio, F., Ollila, D.W., Sartor, C.I., Graham, M.L., and Perou, C.M. (2007). The triple negative paradox: primary tumor chemosensitivity of breast cancer subtypes. Clin. Cancer Res. *13*, 2329–2334.
2. Liedtke, C., Mazouni, C., Hess, K.R., André, F., Tordai, A., Mejia, J.A., Symmans, W.F., Gonzalez-Angulo, A.M., Hennessy, B., Green, M., et al. (2008). Response to neoadjuvant therapy and long-term survival in patients with triple-negative breast cancer. J. Clin. Oncol. *26*, 1275–1281.
3. Carey, L., Winer, E., Viale, G., Cameron, D., and Gianni, L. (2010). Triple-negative breast cancer: disease entity or title of convenience? Nat. Rev. Clin. Oncol. *7*, 683–692.
4. Bianchini, G., Balko, J.M., Mayer, I.A., Sanders, M.E., and Gianni, L. (2016). Triple-negative breast cancer: challenges and opportunities of a heterogeneous disease. Nat. Rev. Clin. Oncol. *13*, 674–690.
5. Bianchini, G., De Angelis, C., Licata, L., and Gianni, L. (2022). Treatment landscape of triple- negative breast cancer - expanded options, evolving needs. Nat. Rev. Clin. Oncol. *19*, 91–113.
6. Denkert, C., Loibl, S., Müller, B.M., Eidtmann, H., Schmitt, W.D., Eiermann, W., Gerber, B., Tesch, H., Hilfrich, J., Huober, J., et al. (2013). Ki67 levels as predictive and prognostic parameters in pretherapeutic breast cancer core biopsies: a translational investigation in the neoadjuvant GeparTrio trial. Ann. Oncol. *24*, 2786–2793.
7. Schmid, P., Cortes, J., Pusztai, L., McArthur, H., Kümmel, S., Bergh, J., Denkert, C., Park, Y.H., Hui, R., Harbeck, N., et al. (2020). Pembrolizumab for Early Triple-Negative Breast Cancer. N. Engl. J. Med. *382*, 810–821.
8. Mittendorf, E.A., Zhang, H., Barrios, C.H., Saji, S., Jung, K.H., Hegg, R., Koehler, A., Sohn, J., Iwata, H., Telli, M.L., et al. (2020). Neoadjuvant atezolizumab in combination with sequential nab-paclitaxel and anthracycline- based chemotherapy versus placebo and chemotherapy in patients with early-stage triple-negative breast cancer (IMpassion031): a randomised, double-blind, phase 3 trial. Lancet *396*, 1090–1100.
9. Cortes, J., Cescon, D.W., Rugo, H.S., Nowecki, Z., Im, S.A., Yusof, M.M., Gallardo, C., Lipatov, O., Barrios, C.H., Holgado, E., et al. (2020). Pembrolizumab plus chemotherapy versus placebo plus chemotherapy for previously untreated locally recurrent inoperable or metastatic triple-negative breast cancer (KEYNOTE-355): a randomised, placebo-controlled, double-blind, phase 3 clinical trial. Lancet *396*, 1817–1828.
10. Schmid, P., Adams, S., Rugo, H.S., Schneeweiss, A., Barrios, C.H., Iwata, H., Diéras, V., Hegg, R., Im, S.A., Shaw Wright, G., et al. (2018). Atezolizumab and Nab-Paclitaxel in Advanced Triple-Negative Breast Cancer. N. Engl. J. Med. *379*, 2108–2121.
11. Cortazar, P., Zhang, L., Untch, M., Mehta, K., Costantino, J.P., Wolmark, N., Bonnefoi, H., Cameron, D., Gianni, L., Valagussa, P., et al. (2014). Pathological complete response and long-term clinical benefit in breast cancer: the CTNeoBC pooled analysis. Lancet *384*, 164–172.
12. Loibl, S., Schneeweiss, A., Huober, J., Braun, M., Rey, J., Blohmer, J.U., Furlanetto, J., Zahm, D.M., Hanusch, C., Thomalla, J., et al. (2022). Neoadjuvant durvalumab improves survival in early triple-negative breast cancer independent of pathological complete response. Ann. Oncol. *33*, 1149–1158.
13. von Minckwitz, G., Blohmer, J.U., Costa, S.D., Denkert, C., Eidtmann, H., Eiermann, W., Gerber, B., Hanusch, C., Hilfrich, J., Huober, J., et al. (2013). Response-guided neoadjuvant chemotherapy for breast cancer. J. Clin. Oncol. *31*, 3623–3630.
14. Loibl, S., Untch, M., Burchardi, N., Huober, J., Sinn, B.V., Blohmer, J.U., Grischke, E.M., Furlanetto, J., Tesch, H., Hanusch, C., et al. (2019). A randomised phase II study investigating durvalumab in addition to an anthracycline taxane-based neoadjuvant therapy in early triple-negative breast cancer: clinical results and biomarker analysis of GeparNuevo study. Ann. Oncol. *30*, 1279–1288.
15. Foldi, J., Silber, A., Reisenbichler, E., Singh, K., Fischbach, N., Persico, J., Adelson, K., Katoch, A., Horowitz, N., Lannin, D., et al. (2021). Neoadjuvant durvalumab plus weekly nab-paclitaxel and dose-dense doxorubicin/cyclophosphamide in triple-negative breast cancer. NPJ Breast Cancer *7*, 9.
16. Blenman, K.R.M., Marczyk, M., Karn, T., Qing, T., Li, X., Gunasekharan, V., Yaghoobi, V., Bai, Y., Ibrahim, E.Y., Park, T., et al. (2022). Predictive Markers of Response to Neoadjuvant Durvalumab with Nab-Paclitaxel and Dose-Dense Doxorubicin/Cyclophosphamide in Basal-Like Triple-Negative Breast Cancer. Clin. Cancer Res. *28*, 2587–2597.
17. Geyer, C.E., Sikov, W.M., Huober, J., Rugo, H.S., Wolmark, N., O'Shaughnessy, J., Maag, D., Untch, M., Golshan, M., Lorenzo, J.P., et al. (2022). Long-term efficacy and safety of addition of carboplatin with or without veliparib to standard neoadjuvant chemotherapy in triple-negative breast cancer: 4-year follow-up data from BrighTNess, a randomized phase III trial. Ann. Oncol. *33*, 384–394.
18. Filho, O.M., Stover, D.G., Asad, S., Ansell, P.J., Watson, M., Loibl, S., Geyer, C.E., Jr., Bae, J., Collier, K., Cherian, M., et al. (2021). Association of Immunophenotype With Pathologic Complete Response to Neoadjuvant Chemotherapy for Triple-Negative Breast Cancer: A Secondary Analysis of the BrighTNess Phase 3 Randomized Clinical Trial. JAMA Oncol. *7*, 603–608.
19. Liberzon, A., Birger, C., Thorvaldsdóttir, H., Ghandi, M., Mesirov, J.P., and Tamayo, P. (2015). The Molecular Signatures Database (MSigDB) hallmark gene set collection. Cell Syst. *1*, 417–425.
20. Blank, C.U., Rozeman, E.A., Fanchi, L.F., Sikorska, K., van de Wiel, B., Kvistborg, P., Krijgsman, O., van den Braber, M., Philips, D., Broeks, A., et al. (2018). Neoadjuvant versus adjuvant ipilimumab plus nivolumab in macroscopic stage III melanoma. Nat. Med. *24*, 1655–1661.
21. Patel, S.P., Othus, M., Chen, Y., Wright, G.P., Jr., Yost, K.J., Hyngstrom, J.R., Hu-Lieskovan, S., Lao, C.D., Fecher, L.A., Truong, T.G., et al. (2023). Neoadjuvant-Adjuvant or Adjuvant-Only Pembrolizumab in Advanced Melanoma. N. Engl. J. Med. *388*, 813–823.
22. Liu, J., Blake, S.J., Yong, M.C.R., Harjunpää, H., Ngiow, S.F., Takeda, K., Young, A., O'Donnell, J.S., Allen, S., Smyth, M.J., and Teng, M.W.L. (2016). Improved Efficacy of Neoadjuvant Compared to Adjuvant Immunotherapy to Eradicate Metastatic Disease. Cancer Discov. *6*, 1382–1399.
23. Sharma, P., Stecklein, S.R., Yoder, R., Staley, J.M., Schwensen, K., O'Dea, A., Nye, L.E., Elia, M., Satelli, D., Crane, G., et al. (2022). Clinical and biomarker results of neoadjuvant phase II study of pembrolizumab and carboplatin plus docetaxel in triple-negative breast cancer (TNBC) (NeoPACT). Clinical Trial. JAMA Oncol. *10*, 227–235.
24. Denkert, C., von Minckwitz, G., Brase, J.C., Sinn, B.V., Gade, S., Kronenwett, R., Pfitzner, B.M., Salat, C., Loi, S., Schmitt, W.D., et al. (2015). Tumor-infiltrating lymphocytes and response to neoadjuvant chemotherapy with or without carboplatin in human epidermal growth factor receptor 2-positive and triple-negative primary breast cancers. J. Clin. Oncol. *33*, 983–991.
25. Tawbi, H.A., Schadendorf, D., Lipson, E.J., Ascierto, P.A., Matamala, L., Castillo Gutiérrez, E., Rutkowski, P., Gogas, H.J., Lao, C.D., De Menezes, J.J., et al. (2022). Relatlimab and Nivolumab versus Nivolumab in Untreated Advanced Melanoma. N. Engl. J. Med. *386*, 24–34.
26. Larkin, J., Chiarion-Sileni, V., Gonzalez, R., Grob, J.J., Rutkowski, P., Lao, C.D., Cowey, C.L., Schadendorf, D., Wagstaff, J., Dummer, R., et al. (2019). Five-Year Survival with Combined Nivolumab and Ipilimumab in Advanced Melanoma. N. Engl. J. Med. *381*, 1535–1546.

CellPress
OPEN ACCESS

27. Overman, M.J., Lonardi, S., Wong, K.Y.M., Lenz, H.J., Gelsomino, F., Aglietta, M., Morse, M.A., Van Cutsem, E., McDermott, R., Hill, A., et al. (2018). Durable Clinical Benefit With Nivolumab Plus Ipilimumab in DNA Mismatch Repair- Deficient/Microsatellite Instability-High Metastatic Colorectal Cancer. J. Clin. Oncol. *36*, 773–779.
28. Motzer, R.J., Tannir, N.M., McDermott, D.F., Arén Frontera, O., Melichar, B., Choueiri, T.K., Plimack, E.R., Barthélémy, P., et al. (2018). Nivolumab plus Ipilimumab versus Sunitinib in Advanced Renal-Cell Carcinoma. N. Engl. J. Med. *378*, 1277–1290.
29. Takahara, K., Kessler, E., Biniaminov, L., Brusel, M., Eddy, R.L., Jani-Sait, S., Shows, T.B., and Greenspan, D.S. (1994). Type I procollagen COOH--terminal proteinase enhancer protein: identification, primary structure, and chromosomal localization of the cognate human gene (PCOLCE). J. Biol. Chem. *269*, 26280–26285.
30. Xiang, A., Lin, X., Xu, L., Chen, H., Guo, J., and Zhou, F. (2020). PCOLCE Is Potent Prognostic Biomarker and Associates With Immune Infiltration in Gastric Cancer. Front. Mol. Biosci. *7*, 544895.
31. Newman, A.C., Nakatsu, M.N., Chou, W., Gershon, P.D., and Hughes, C.C.W. (2011). The requirement for fibroblasts in angiogenesis: fibroblast-derived matrix proteins are essential for endothelial cell lumen formation. Mol. Biol. Cell *22*, 3791–3800.
32. Orimo, A., Gupta, P.B., Sgroi, D.C., Arenzana-Seisdedos, F., Delaunay, T., Naeem, R., Carey, V.J., Richardson, A.L., and Weinberg, R.A. (2005). Stromal fibroblasts present in invasive human breast carcinomas promote tumor growth and angiogenesis through elevated SDF-1/CXCL12 secretion. Cell *121*, 335–348.
33. Timperi, E., Gueguen, P., Molgora, M., Magagna, I., Kieffer, Y., Lopez-Lastra, S., Sirven, P., Baudrin, L.G., Baulande, S., Nicolas, A., et al. (2022). Lipid-Associated Macrophages Are Induced by Cancer-Associated Fibroblasts and Mediate Immune Suppression in Breast Cancer. Cancer Res. *82*, 3291–3306.
34. Nicolas, A.M., Pesic, M., Engel, E., Ziegler, P.K., Diefenhardt, M., Kennel, K.B., Buettner, F., Conche, C., Petrocelli, V., Elwakeel, E., et al. (2022). Inflammatory fibroblasts mediate resistance to neoadjuvant therapy in rectal cancer. Cancer Cell *40*, 168–184.e13.
35. Kieffer, Y., Hocine, H.R., Gentric, G., Pelon, F., Bernard, C., Bourachot, B., Lameiras, S., Albergante, L., Bonneau, C., Guyard, A., et al. (2020). Single-Cell Analysis Reveals Fibroblast Clusters Linked to Immunotherapy Resistance in Cancer. Cancer Discov. *10*, 1330–1351.
36. Costa, A., Kieffer, Y., Scholer-Dahirel, A., Pelon, F., Bourachot, B., Cardon, M., Sirven, P., Magagna, I., Fuhrmann, L., Bernard, C., et al. (2018). Fibroblast Heterogeneity and Immunosuppressive Environment in Human Breast Cancer. Cancer Cell *33*, 463–479.e10.
37. Friedman, G., Levi-Galibov, O., David, E., Bornstein, C., Giladi, A., Dadiani, M., Mayo, A., Halperin, C., Pevsner-Fischer, M., Lavon, H., et al. (2020). Cancer-associated fibroblast compositions change with breast cancer progression linking the ratio of S100A4$^+$ and PDPN$^+$ CAFs to clinical outcome. Nat. Can. (Ott.) *1*, 692–708.
38. Bartoschek, M., Oskolkov, N., Bocci, M., Lövrot, J., Larsson, C., Sommarin, M., Madsen, C.D., Lindgren, D., Pekar, G., Karlsson, G., et al. (2018). Spatially and functionally distinct subclasses of breast cancer-associated fibroblasts revealed by single cell RNA sequencing. Nat. Commun. *9*, 5150.
39. Pelon, F., Bourachot, B., Kieffer, Y., Magagna, I., Mermet-Meillon, F., Bonnet, I., Costa, A., Givel, A.M., Attieh, Y., Barbazan, J., et al. (2020). Cancer-associated fibroblast heterogeneity in axillary lymph nodes drives metastases in breast cancer through complementary mechanisms. Nat. Commun. *11*, 404.
40. Chung, H.C., Saada-Bouzid, E., Muñoz, F.L., Yanez, E., Im, S.-A., Castanon, E., Graham, D.M., Garcia-Corbacho, J., Lopez, J., Ghori, R., et al. (2020). Lenvatinib plus pembrolizumab for previously treated, advanced triple-negative breast cancer: Early results from the multicohort phase 2 LEAP-005 study. Cancer *81*. Abstract nr PS12-07.
41. Saba, N.F., Steuer, C.E., Ekpenyong, A., McCook-Veal, A., Magliocca, K., Patel, M., Schmitt, N.C., Stokes, W., Bates, J.E., Rudra, S., et al. (2023). Pembrolizumab and cabozantinib in recurrent metastatic head and neck squamous cell carcinoma: a phase 2 trial. Nat. Med. *29*, 880–887.
42. Guo, Y., Guo, J., Cheng, Y., Wang, Z., Li, Y., Lv, D., Yin, Y., Li, G., Wu, L., Huang, Y., et al. (2023). Phase Ib/IIa safety and efficacy of PM8002, a bispecific antibody targeting PD-L1 and VEGF-A, as a monotherapy in patients with advanced solid tumors. J. Clin. Oncol. *41*, 2536.
43. Zhao, Y., Chen, G., Chen, J., Zhuang, L., Du, Y., Yu, Q., Zhuang, W., Zhao, Y., Zhou, M., Zhang, W., et al. (2023). AK112, a novel PD-1/VEGF bispecific antibody, in combination with chemotherapy in patients with advanced non-small cell lung cancer (NSCLC): an open-label, multicenter, phase II trial. EClinicalMedicine *62*, 102106.
44. Tzuri, N., Yegodayev, K.M., Novoplansky, O., Elkabets, M., Aharoni, A., and Papo, N. (2023). Developing a dual VEGF/PDL1 inhibitor based on high-affinity scFv heterodimers as an anti-cancer therapeutic strategy. Sci. Rep. *13*, 11923.
45. Brueffer, C., Gladchuk, S., Winter, C., Vallon-Christersson, J., Hegardt, C., Häkkinen, J., George, A.M., Chen, Y., Ehinger, A., Larsson, C., et al. (2020). The mutational landscape of the SCAN-B real-world primary breast cancer transcriptome. EMBO Mol. Med. *12*, e12118.
46. Subramanian, A., Tamayo, P., Mootha, V.K., Mukherjee, S., Ebert, B.L., Gillette, M.A., Paulovich, A., Pomeroy, S.L., Golub, T.R., Lander, E.S., and Mesirov, J.P. (2005). Gene set enrichment analysis: a knowledge-based approach for interpreting genome-wide expression profiles. Proc. Natl. Acad. Sci. USA *102*, 15545–15550.
47. Staaf, J., Glodzik, D., Bosch, A., Vallon-Christersson, J., Reuterswärd, C., Häkkinen, J., Degasperi, A., Amarante, T.D., Saal, L.H., Hegardt, C., et al. (2019). Whole-genome sequencing of triple-negative breast cancers in a population-based clinical study. Nat. Med. *25*, 1526–1533.
48. Hudis, C.A., Barlow, W.E., Costantino, J.P., Gray, R.J., Pritchard, K.I., Chapman, J.A.W., Sparano, J.A., Hunsberger, S., Enos, R.A., Gelber, R.D., and Zujewski, J.A. (2007). Proposal for standardized definitions for efficacy end points in adjuvant breast cancer trials: the STEEP system. J. Clin. Oncol. *25*, 2127–2132.
49. Sinn, B.V., Loibl, S., Hanusch, C.A., Zahm, D.M., Sinn, H.P., Untch, M., Weber, K., Karn, T., Becker, C., Marmé, F., et al. (2021). Immune-related Gene Expression Predicts Response to Neoadjuvant Chemotherapy but not Additional Benefit from PD-L1 Inhibition in Women with Early Triple-negative Breast Cancer. Clin. Cancer Res. *27*, 2584–2591.
50. Robinson, M.D., McCarthy, D.J., and Smyth, G.K. (2010). edgeR: a Bioconductor package for differential expression analysis of digital gene expression data. Bioinformatics *26*, 139–140.

STAR★METHODS

KEY RESOURCES TABLE

REAGENT or RESOURCE	SOURCE	IDENTIFIER
Biological samples		
Tumor biopsies and data from the neoadjuvant GeparNuevo trial	Loibl et al.[12]	NCT02685059
Tissue microarray from GeparNuevo core biopsies	GBG biobank at Institute of Pathology, Philipps University Marburg	NCT02685059
Data generated from tumor biopsies from the neoadjuvant MEDI4736 trial	Blenman et al.[16]	NCT02489448
Data generated from tumor biopsies from the BrighTNess trial	Filho et al.[18]	NCT02032277
Data generated from tumor bioosies from the Sweden Cancerome Analysis Network-Breast (SCAN-B) population-based cohort	Brueffer et al.[45]	NCT02306096
Critical commercial assays		
Oncology Biomarker mRNA Panel, ILM Kit, 1x96	HTG Molecular Diagnostics, Tucson, Arizona, US	916-002-096
Deposited data		
Data for this study	GitHub	https://github.com/tkarn/G9-HTG
Analysis code	Zenodo	https://doi.org/10.5281/zenodo.13833943
Output files	GitHub	https://github.com/tkarn/G9-HTG
Software and algorithms		
R version 4.2.1	The R Project for Statistical Computing	https://www.r-project.org
GSEA software, version 4.2.3	Subramanian et al.[46]	https://www.gsea-msigdb.org/gsea/downloads.jsp
The Molecular Signatures Database (MSigDB	Liberzon et al.[19]	https://www.gsea-msigdb.org/gsea/msigdb

EXPERIMENTAL MODEL AND STUDY PARTICIPANT DETAILS

Study design and clinical cohorts

The neoadjuvant phase 2 double-blind randomized placebo-controlled GeparNuevo trial (NCT02685059) included 174 patients with cT1-cT4a-d triple-negative breast cancer (TNBC) who received durvalumab 1.5 g or placebo every 4 weeks added to nab-paclitaxel 125 mg/m^2 body surface area (BSA) weekly for 12 weeks, followed by durvalumab vs. placebo every 4 weeks plus epirubicin 90 mg/m^2 BSA/cyclophosphamide 600 mg/m^2 BSA every 2 weeks followed by surgery.

A subset of patients received one injection of durvalumab vs. placebo alone in the window phase 2 weeks before start of nab-paclitaxel. Based on the recommendation of the Independent Data Monitoring Committee (IDMC), the window phase was stopped after 117 patients were recruited. Thereafter all patients started with durvalumab/placebo plus chemotherapy on day 1. Durvalumab was not continued after surgery. For longitudinal biomarker analyses, formalin-fixed, paraffin-embedded (FFPE) tumor samples were collected before the start of therapy (A-samples), after the window phase (B-samples) and after 12 weeks of nab-paclitacel +/− durvalumab (C-samples). Tumor samples were sent to the biobank prior to randomization for central testing of expression of human epidermal growth factor receptor 2 (HER2), estrogen receptor (ER), progesterone receptor (PR) and Ki-67 and assessment of tumor-infiltrating lymphocytes (TILs). Samples were stored in the central biobank for gene expression analysis. The analysis set for biomarker evaluation included the modified intent to treat (mITT) analysis set from the GeparNuevo study (n = 174; durvalumab: n = 88; placebo: n = 86).

The clinical trial was approved by the relevant ethics committee and authorities, informed consent was obtained for clinical trial participation, biomaterial collection as well as translational investigations. In addition, the translational investigations were approved by the ethics committee of the Philipps University Marburg (38/20 and 121/20).

As validation cohorts, the neoadjuvant MEDI4736 trial (NCT02489448) as well as the BrighTNess trial (NCT02032277) were used for validation of genes related to pCR. RNA-Seq data and pCR status from the MEDI4736-trial was available for 55 patients.[15] RNA-Seq data from BrighTNess was available for 482 patients.[17] For additional validations regarding survival, RNA-Seq data

of 326 TNBC samples from the Sweden Cancerome Analysis Network-Breast (SCAN-B; NCT02306096) population-based cohort was used.[47,45]

METHOD DETAILS

Endpoints

Pathological complete response (pCR) was defined as no invasive and no non-invasive residuals in breast and lymph nodes (ypT0/ypN0). Distant disease-free survival (DDFS) was defined as time in months from randomization until any distant recurrence, any secondary malignancy or death due to any cause, whichever occurred first.[48] Patients without event were censored at the date of the last contact.

Gene expression analysis using the HTG OBP panel

For gene expression analysis in FFPE core biopsies, two different approaches were used. For longitudinal gene expression the HTG EdgeSeq Oncology Biomarker Panel (OBP, HTG Molecular Diagnostics, Tucson, Arizona, US) was used, as described before.[49] One single FFPE section with a minimum of 20% tumor cellularity was used for gene expression analysis, which makes this approach suitable for longitudinal evaluations in the neoadjuvant setting.

The HTG EdgeSeq Oncology Biomarker Panel measures the expression of 2549 genes related to tumor biology. For quality control, we transformed the reads to counts permillion (CPM)[50] and calculated the mean of four negative and four positive internal controls for each sample. We repeated processing for a sample if the mean of its positive controls was below two standard deviations (SDs) of the grand mean across all samples or if the mean of its negative controls were above two SDs from the grand mean.[47] As additional quality control, samples with less than 1.5 million total counts were excluded from further analysis. To improve reproducibility a modification of the CPM method was applied by introducing a lower bound of 3 on CPM values. Valid QC-cleaned HTG data was available in $n = 148$ patients of the mITT analysis set (durvalumab: $n = 77$; placebo: $n = 71$). Gene expression analysis was performed using R version 4.2.1.

Analysis of cellular pathways based on temporal expression data

For gene set enrichment analysis (GSEA), the 2549 genes of the HTG OBP panel were mapped to 25 gene groups and pathways predefined by HTG (HTG gene sets), as well as to the established 50 hallmark gene sets (HM) of the Human Molecular Signatures Database[19] representing well-defined biological states and processes. For several genes in the GSEA datasets, there was more than one associated gene set, so that the number of gene-gene set allocations was higher than the number of genes in the HTG OBP panel.

The GSEA was performed with the GSEAPreranked analysis of the GSEA software, version 4.2.3[46] by using a rank metric based on the respective *p*-values and the sign of the test statistic of univariate regression analyses for pCR and DDFS in different cohorts (-log10(*p*-value)*sgn(test statistic) for pCR; log10(*p*-value)*sgn(test statistic) for DDFS). Positive values of the rank metric refer to genes that are more likely upregulated in patients with a pCR resp. with a longer survival, negative values to genes that are more likely downregulated.

The following GSEA statistics were generated: Enrichment score (ES) for each significant gene set: degree to which gene set is overrepresented at the top or bottom of the ranked list of genes in this gene set; normalized enrichment score (NES): normalized across analyzed gene sets; *p*-value for the statistical significance of the ES; false discovery rate (FDR): probability that the NES represents a false positive finding. The threshold for significant enrichment were defined as a *p*-value of <0.05 and an FDR of <0.25. For analysis of more than one outcome parameter (e.g., combined GSEA for pCR and DDFS), only those gene sets were included that had a significant enrichment for at least one of the two outcome parameters.

Gene set groups (GSG) and gene class assignment

For some evaluations, gene sets with similar biological functions were combined to gene set groups (GSGs). The combined *immune* GSG was defined as those genes included in the gene sets immuno-oncology (HTG), allograft rejection (HM), interferon alpha response (HM) or interferon gamma response (HM). The combined *proliferation*-related GSG was defined as those genes included in the gene sets cell cycle (HTG), E2F targets (HM), G2M checkpoint (HM) or mitotic spindle (HM). The combined *stromal* GSG was defined as those genes included in the gene sets angiogenesis (HTG), angiogenesis (HM), coagulation (HM), epithelial mesenchymal transition (HM), fatty acid metabolism (HM) or myogenesis (HM). The combined *DNA repair* GSG was defined as those genes included in the gene sets DNA repair (HTG) or DNA repair (HM). The *stem cell* GSG was based on the gene set stem cells (HTG).

If genes that are members of several GSGs need to be assigned to a unique class (e.g., for color coding in plots), we used the following ranking of GSGs: 1) *immune*, 2) *proliferation*, 3) *stromal*, 4) *DNA repair*, 5) *stem cell*, 6) *other* (not in any of the above gene sets). Detailed code is available in the SETUP section of the supplemental R script. Figures were created using a licensed version of biorender.com.

Predictive gene signatures

We also defined three predictive gene signatures based on GSGs and response data in the GeparNuevo durvalumab arm (Figures 7E–7H and S8): For *immune* and *proliferation* GSGs, all genes positively predictive for pCR (OR>1 and $p \leq 0.05$) were

included, for the *stromal* GSG, all genes negatively predictive for pCR (OR<1 and $p \leq 0.05$). The signature scores were calculated as unweighted mean of the scaled $\log_2$ values of the respective genes, and zero was used as a cutoff for categorical classification in both finding and validation cohort. The respective gene lists are given in Table S2. Detailed code is available in the supplemental R script.

QUANTIFICATION AND STATISTICAL ANALYSIS

Statistical analysis

All categorical variables were summarized as number and percent of patients in each category. Statistical tests (Wilcoxon signed rank test for longitudinal data) were by default 2-sided, significance level was set to $\alpha = 0.05$. Confidence intervals (CI) symmetrically span 95%. Univariate logistic regression analyses were used for pCR to report odds ratios (OR) with 95% CI. Univariate Cox proportional hazards model were used for DDFS to report hazard ratios with 95% CI.

Correction for multiple testing

The GSEA analysis was adjusted for multiple testing according to the false discovery rate, while all other *p*-values were not adjusted for multiple testing as the study was designed to generate hypothesis for future research rather than to confirm specific hypotheses.

Organization of the bioinformatical analysis

The different analysis teams were physically separated and blinded. Members of the bioinformatic and writing team (CD, TK, AH) are fully blinded during the whole analysis to the clinical data of the GeparNuevo patients. The correlation of HTG gene expression data with clinical outcome and GSEA was performed by the statistics team at GBG headquarter (JR, KW) and the results for all genes transferred on a cohort level to the bioinformatic team with no clinical patient level data. The respective dataset is available in online on GitHub together with the analysis code (see above in section resource availability for links). For additional studies in the GeparNuevo dataset, R scripts were provided to the GBG statistics team and the output figures sent back to the analysis and writing team.

Cell Reports Medicine, Volume *5*

Supplemental information

Molecular adaptation to neoadjuvant immunotherapy in triple-negative breast cancer

Carsten Denkert, Andreas Schneeweiss, Julia Rey, Thomas Karn, Akira Hattesohl, Karsten E. Weber, Sivaramakrishna Rachakonda, Michael Braun, Jens Huober, Paul Jank, Hans-Peter Sinn, Dirk-Michael Zahm, Bärbel Felder, Claus Hanusch, Julia Teply-Szymanski, Frederik Marmé, Tanja Fehm, Jörg Thomalla, Bruno V. Sinn, Thorsten Stiewe, Michal Marczyk, Jens-Uwe Blohmer, Marion van Mackelenbergh, Christian Schem, Peter Staib, Theresa Link, Volkmar Müller, Elmar Stickeler, Daniel G. Stover, Christine Solbach, Otto Metzger-Filho, Christian Jackisch, Charles E. Geyer Jr., Peter A. Fasching, Lajos Pusztai, Valentina Nekljudova, Michael Untch, and Sibylle Loibl

Molecular adaptation to neoadjuvant immunotherapy in triple-negative breast cancer

Denkert et al.

Supplemental data

Table S1: Description of the baseline characteristics of the GeparNuevo biomarker cohort (n=148). Related to Figure 1.

Parameter	*Category*	*Durvalumab N=77 N(%)*	*Placebo N=71 N(%)*	*Overall N=148 N(%)*	*p-value*
Age, years	<40	24 (31.2)	16 (22.5)	40 (27.0)	0.237
	>=40	53 (68.8)	55 (77.5)	108 (73.0)	
Window arm	window	52 (67.5)	44 (62.0)	96 (64.9)	0.496
	no window	25 (32.5)	27 (38.0)	52 (35.1)	
Menopausal Status	premenopausal	45 (58.4)	41 (57.7)	86 (58.1)	1.000
	postmenopausal	32 (41.6)	30 (42.3)	62 (41.9)	
cT	cT1-2	70 (90.9)	68 (95.8)	138 (93.2)	0.239
	cT3-4	7 (9.1)	3 (4.2)	10 (6.8)	
cN by sonography	cN0	51 (67.1)	50 (70.4)	101 (68.7)	0.665
	cN+	25 (32.9)	21 (29.6)	46 (31.3)	
	missing	1	0	1	
Breast Cancer Stage (binary)	Stage 0 or I	27 (35.1)	26 (36.6)	53 (35.8)	0.865
	Stage IIA and higher	50 (64.9)	45 (63.4)	95 (64.2)	
Grading	G2	13 (16.9)	13 (18.3)	26 (17.6)	0.820
	G3	64 (83.1)	58 (81.7)	122 (82.4)	
Histological tumor type	ductal or ductal-lobular invasive	60 (77.9)	58 (81.7)	118 (79.7)	0.437
	lobular invasive	0 (0.0)	1 (1.4)	1 (0.7)	
	other	17 (22.1)	12 (16.9)	29 (19.6)	
sTILs	low (0-10%)	27 (35.1)	26 (36.6)	53 (35.8)	0.852
	intermediate (11-59%)	39 (50.6)	33 (46.5)	72 (48.6)	
	high (60-100%)	11 (14.3)	12 (16.9)	23 (15.5)	
PD-L1	negative	8 (11.3)	10 (14.9)	18 (13.0)	0.616
	positive	63 (88.7)	57 (85.1)	120 (87.0)	
	missing	6	4	10	
pCR (ypT0 ypN0)	no	35 (45.5)	35 (49.3)	70 (47.3)	0.742
	yes	42 (54.5)	36 (50.7)	78 (52.7)	

p: Fisher's exact test resp. Pearson's Chi-squared test

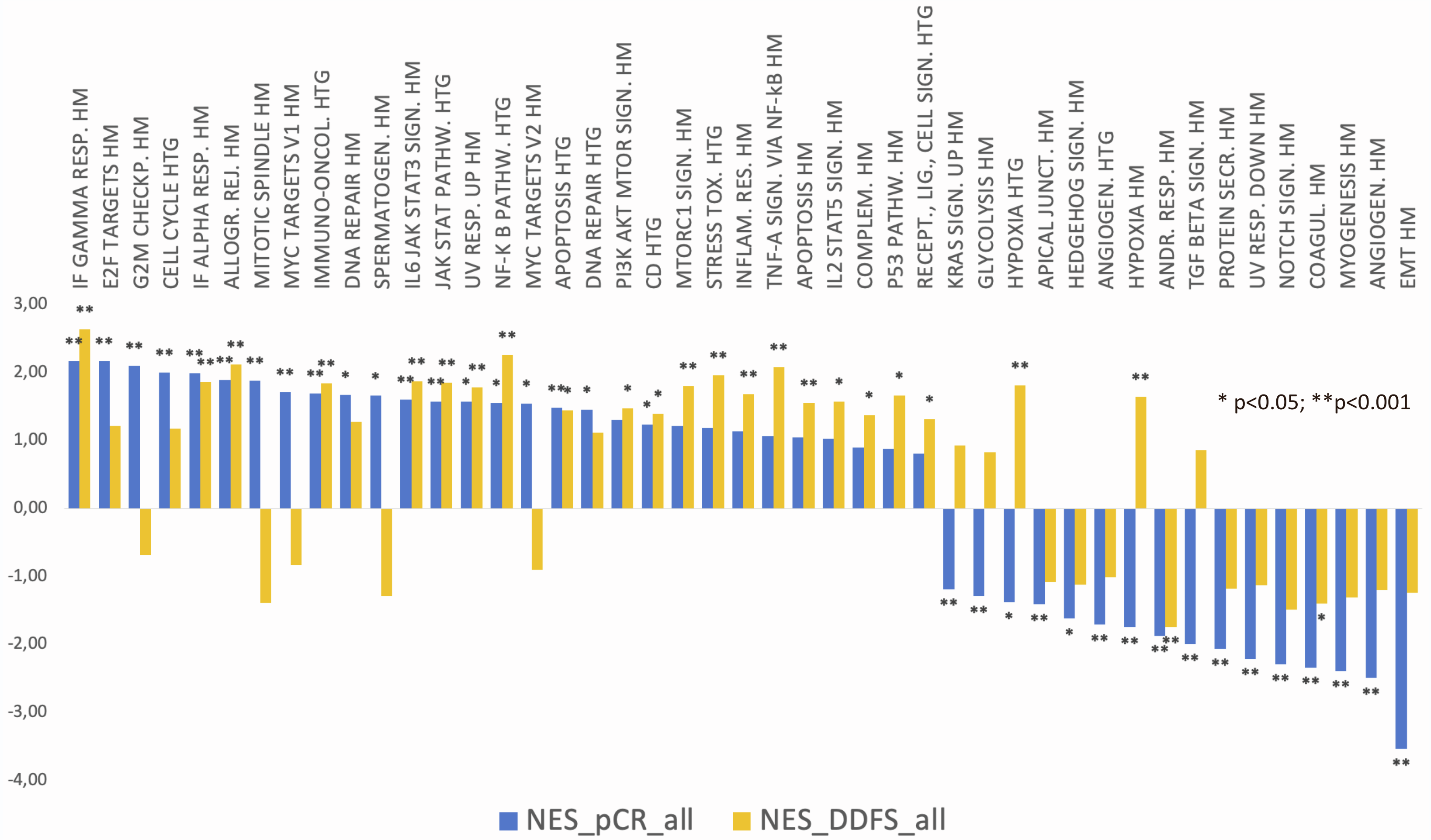

Figure S1: Gene set enrichment analysis for the two clinical endpoints pCR and DDFS (related to figure 2)

Complete G9 cohort: Details of the gene set enrichment analysis based on an analysis of 2549 genes included in the HTG oncology biomarker assay using HTG-defined gene sets (HTG) as well as the Hallmark gene sets (HM). The normalized enrichment score (NES) for pCR (blue) and DDFS (orange) is shown. Only gene sets are included that have a p-value of <0.05 as well as a FDR of <25% for at least one of the endpoints (pCR or DDFS). The analysis shown is based on pretherapeutic core biopsies of the complete cohort, combining both therapy arms.

Complete G9 cohort: genesets for significantly improved DDFS and significantly increased pCR rate

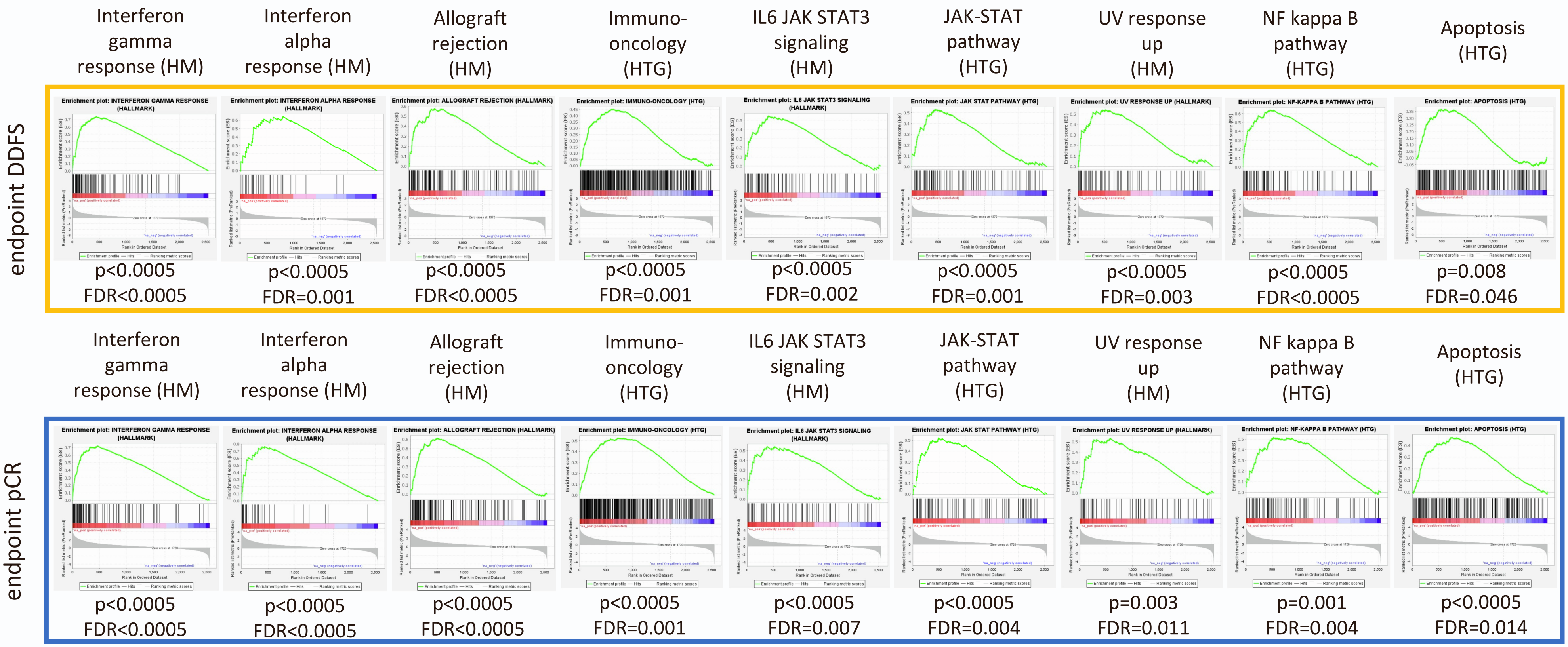

Figure S2: Gene set enrichment analysis for the two clinical endpoints pCR and DDFS (related to figure 2)
Complete G9 cohort: gene sets for significantly improved DDFS and significantly increased pCR rate.

Denkert et al. Figure S3

A Complete G9 cohort: genesets for increased pCR rate without significant effect on DDFS

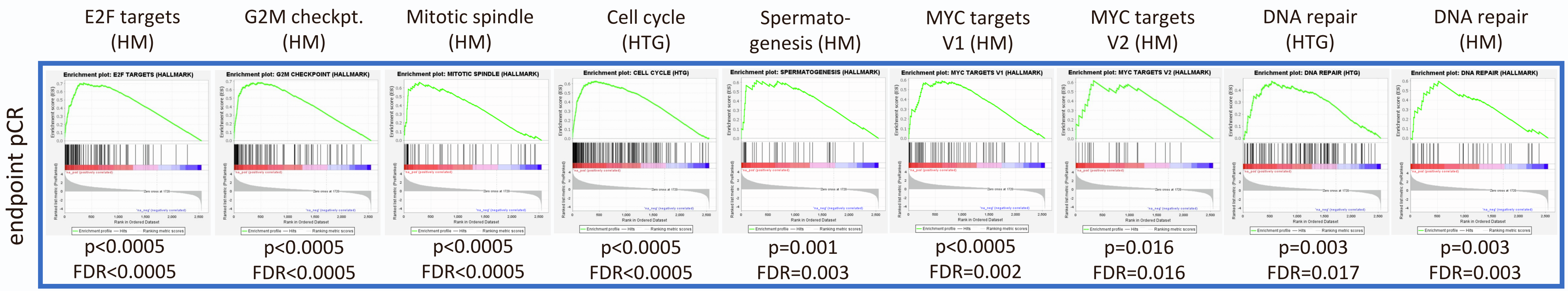

B

Complete G9 cohort: genesets for reduced pCR rate without significant effect on DDFS

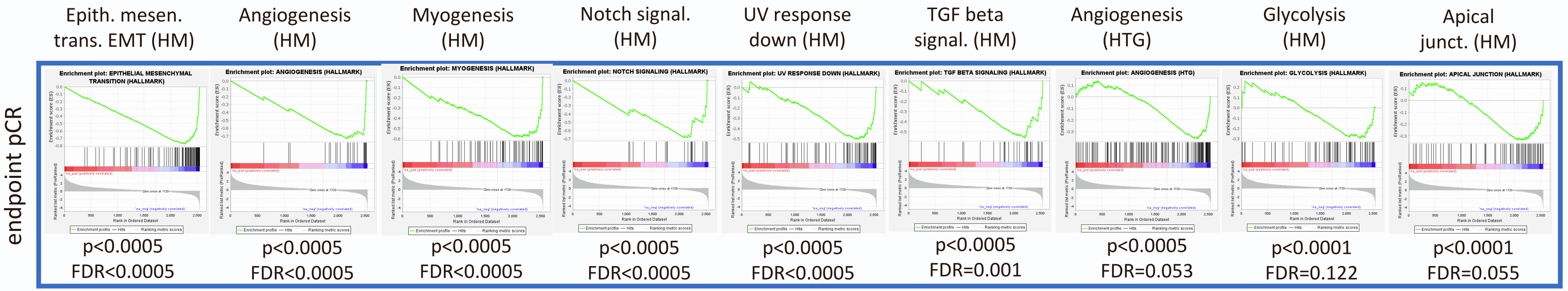

Figure S3: Gene set enrichment analysis for the two clinical endpoints pCR and DDFS (related to figure 2)
(A) Complete G9 cohort: gene sets for increased pCR rate without significant effect on DDFS.
(B) Complete G9 cohort: gene sets for reduced pCR rate without significant effect on DDFS.

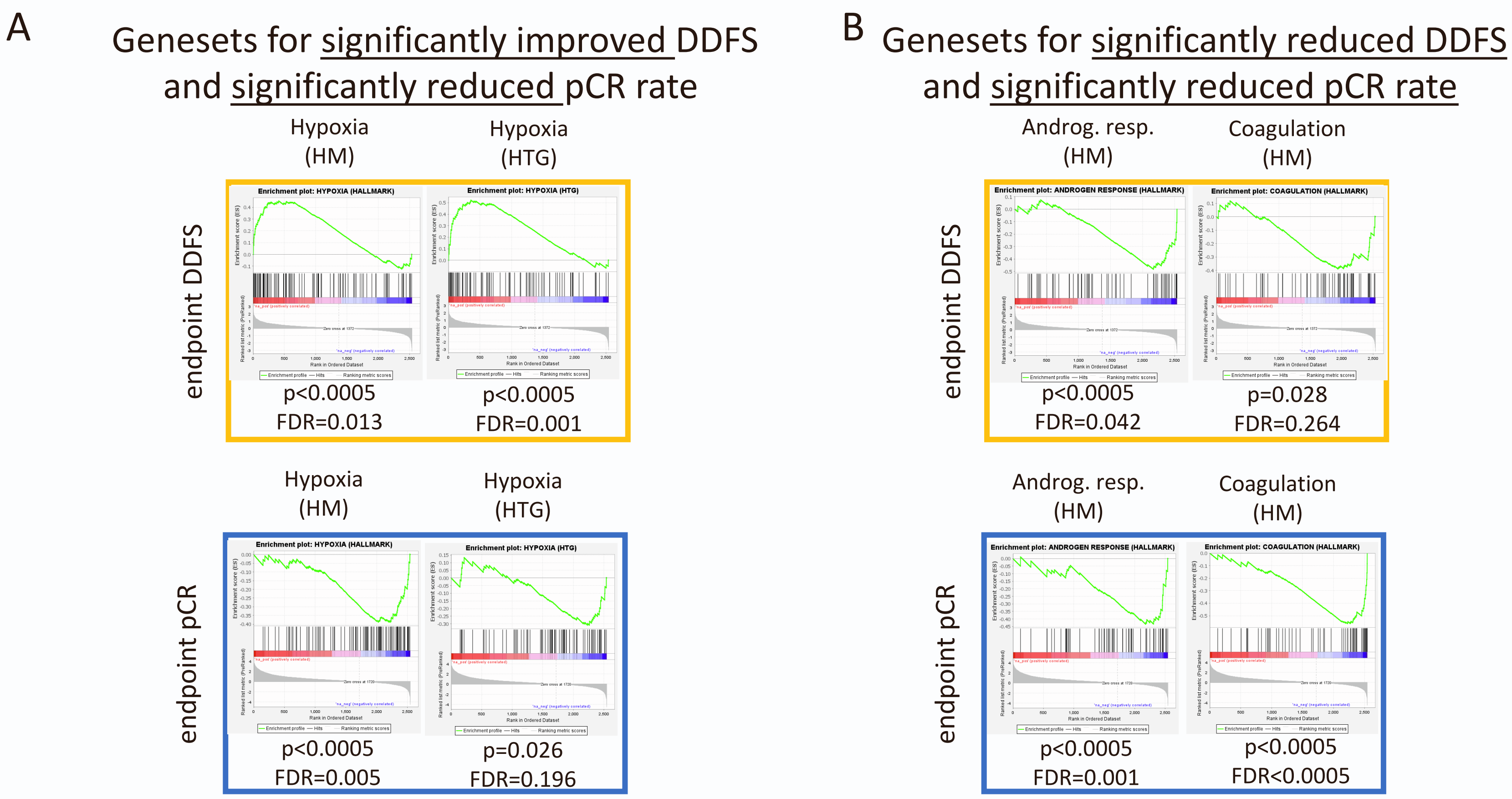

Figure S4: Gene set enrichment analysis for the two clinical endpoints pCR and DDFS (related to figure 2)

(A) Complete G9 cohort: gene sets for significantly improved DDFS and significantly reduced pCR rate.

(B) Complete G9 cohort: gene sets for significantly reduced DDFS and significantly reduced pCR rate.

Denkert et al.

Figure S5

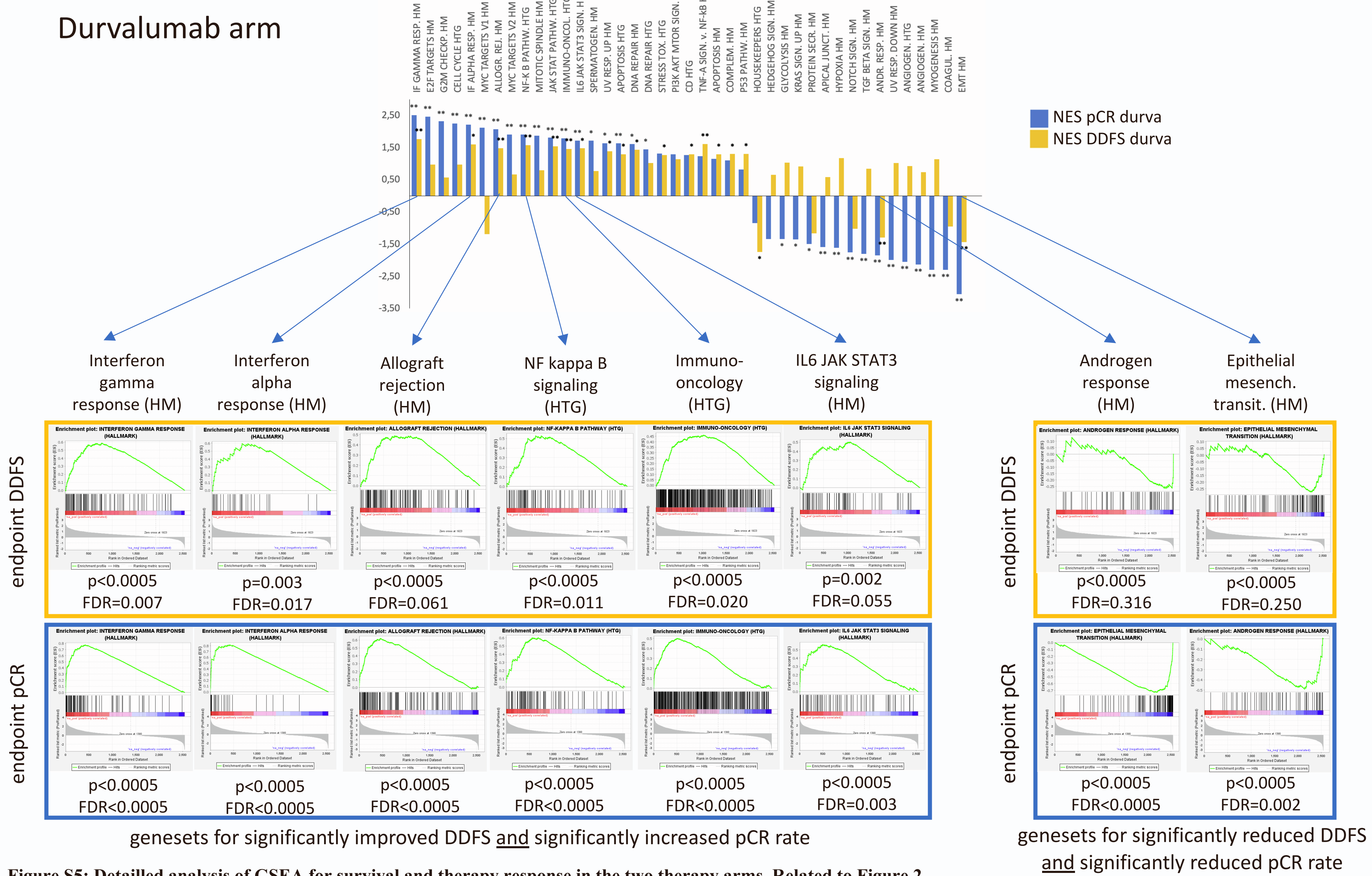

Figure S5: Detailled analysis of GSEA for survival and therapy response in the two therapy arms. Related to Figure 2. Durvalumab arm: Additional details of the analysis shown in Figure 2: GSEA plots for the most important cellular pathways.

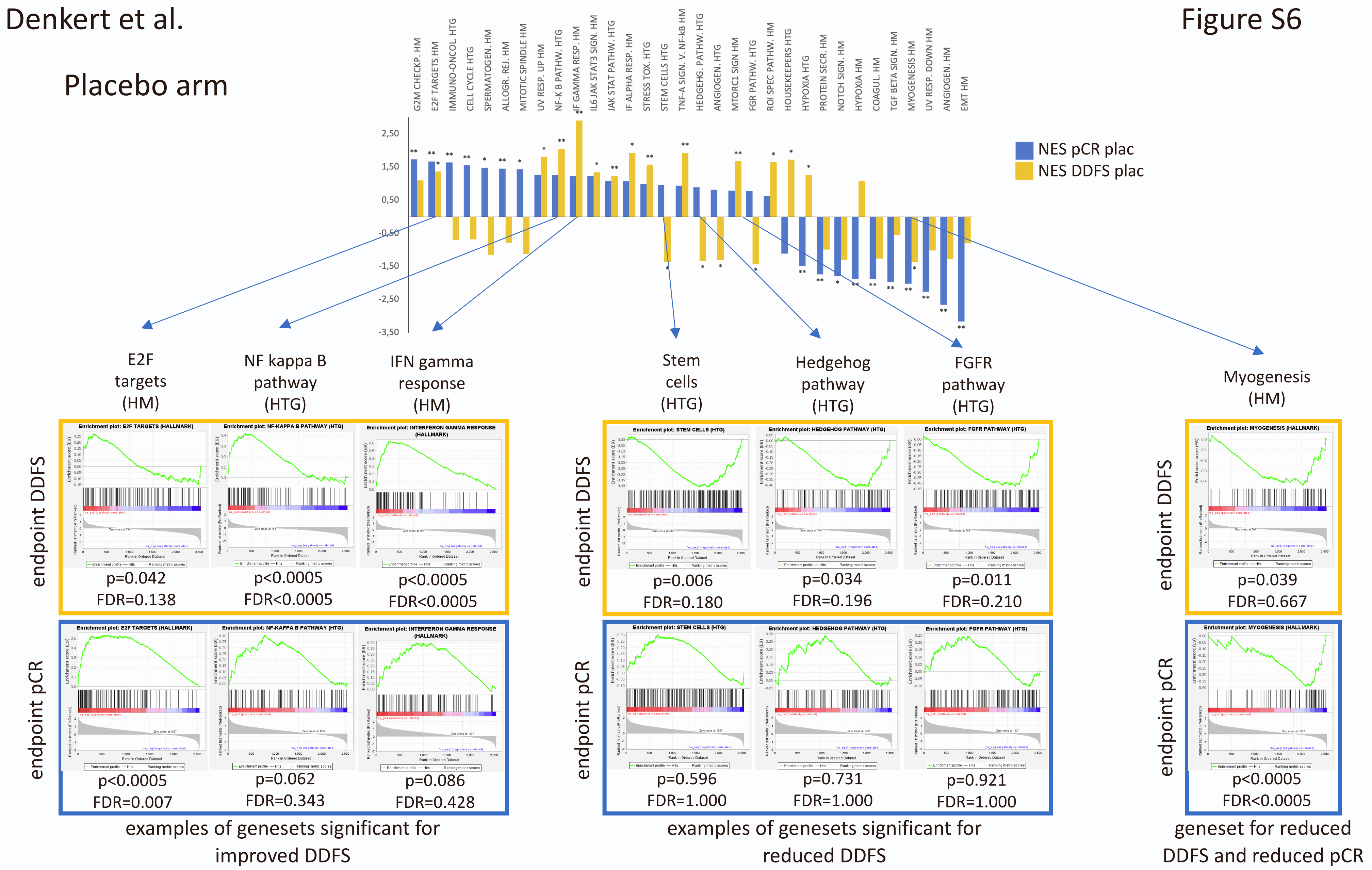

Figure S6: Detailled analysis of GSEA for survival and therapy response in the two therapy arms. Related to Figure 2.
Placebo arm: Additional details of the analysis shown in Figure 2: GSEA plots for the most important cellular pathways.

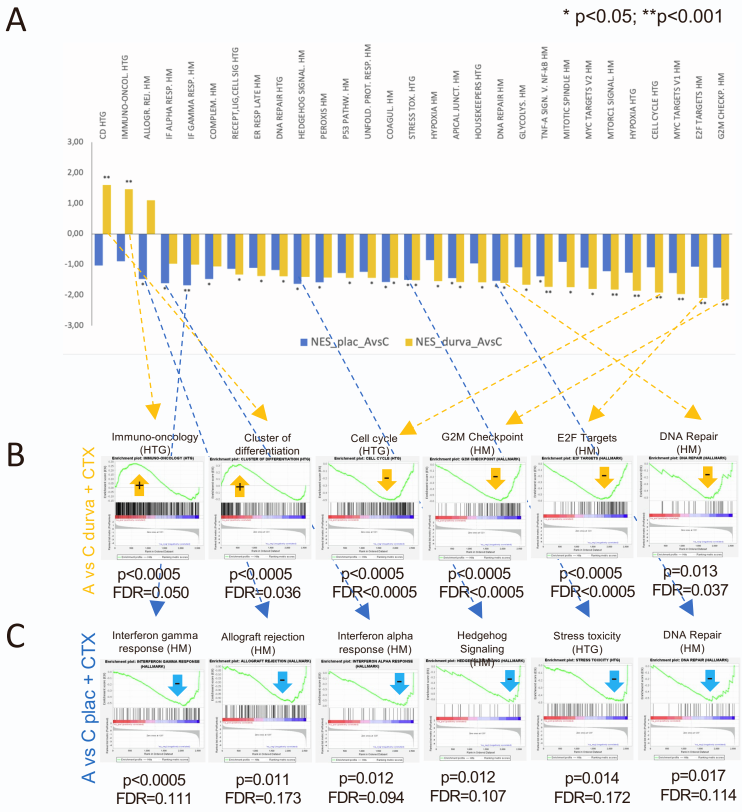

Figure S7: Evaluation of molecular alterations in TNBC before therapy (A-samples) compared with samples after 12 weeks of chemotherapy +/- durvalumab (C-samples). Related to Figure 5.

(**A**) Comparison of gene set enrichment in both therapy arms after 12 weeks of chemotherapy with durvalumab (orange) or without durvalumab (blue). The normalized enrichment score (NES) for placebo (x-axis, blue) and durvalumab (y-axis, orange) is shown.

(B,C) GSEA results for selected gene sets after 12 weeks of chemotherapy with **(B)** or without **(C)** durvalumab. (CTX, chemotherapy; FDR, false discovery rate)

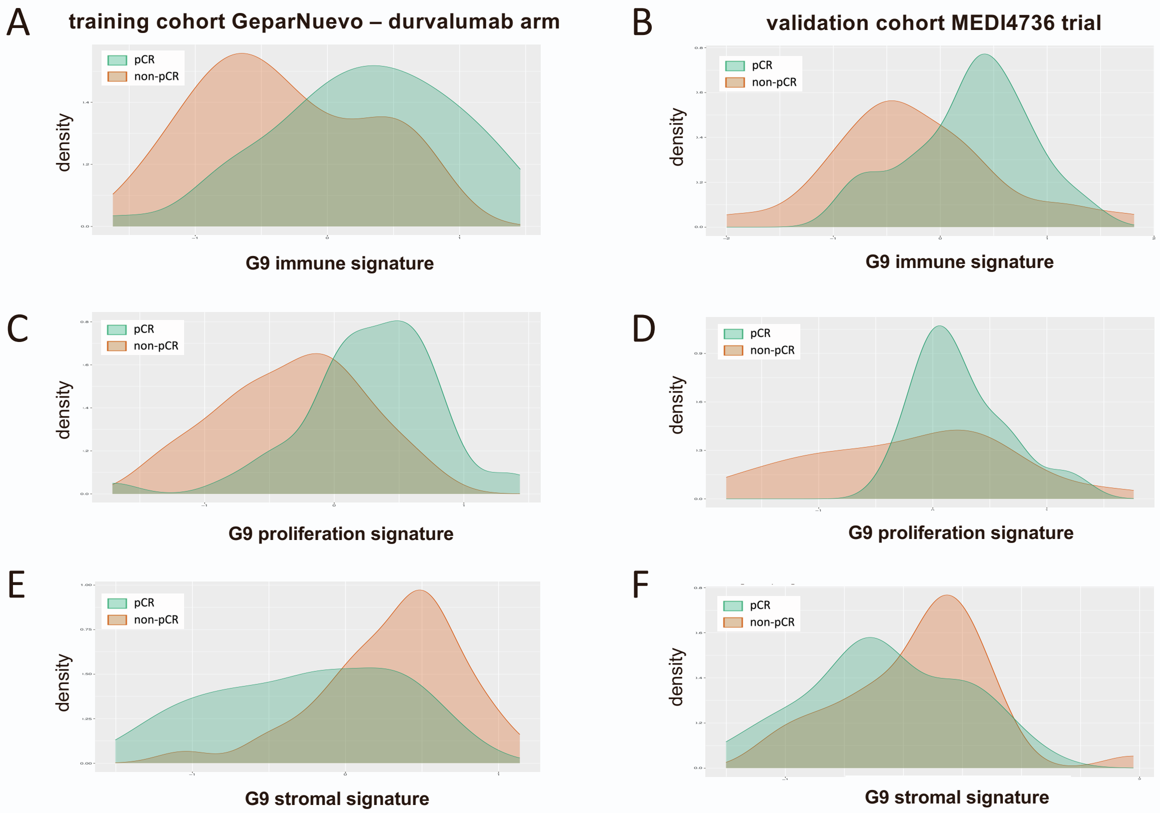

Figure S8: Validation in three independent cohorts (Related to Figure 7). Comparison of distribution of pCR rate for three gene signatures covering immune **(A,B)**, proliferation **(C,D)** and stromal genes **(E,F)** in the GeparNuevo durvalumab training cohort **(A,C,E)** and the validation cohort MEDI4736 **(B,D,F)**.

The negative role of stromal gene expression and the positive role of immune and proliferation genes could be validated in the independent MEDI4736 cohort. For full details see STAR Methods section.

Table S2. Genes included in the stromal, proliferation and immune signature (Related to Figure 7 and Figure S8)

G9.immune.signature.genes	G9.proliferation.signature.genes	G9.stromal.signature.genes
ADAR	ATR	ACTA2
APOL3	AURKA	ANXA1
CA9	AURKB	CALD1
CCL2	BLM	CD55
CCL4	BUB1	COL11A1
CCL5	CASP8AP2	COL1A1
CCL7	CBX5	COL1A2
CD274	CCNA2	COL3A1
CD38	CCNB1	COL5A1
CD47	CCNE1	COL5A2
CD79A	CDC20	COMP
CD86	CDC7	DST
CD8A	CDK2	EDIL3
CDKN2A	CDKN1B	EFNB2
CEBPB	CDKN2A	F3
CHI3L1	CDKN3	FBN1
CTSS	CHEK2	FGF13
CXCL10	CKS2	FLT1
CXCL13	CTPS1	FN1
CXCL9	CUL2	GNG12
CXCR6	DLGAP5	GSN
DDX58	DNMT1	IGFBP4
DHX58	E2F3	IGFBP7
EIF2AK2	ECT2	ITGA2
FGL2	EZH2	ITGAV
GBP1	FBXO5	JAG1
GNLY	GTSE1	KDR
GZMA	HELLS	LOX
GZMB	HIST1H3H	MMP14
HLA-A	HJURP	MMP2
HLA-B	HMGB2	NOTCH4
HLA-E	KIF2C	PCOLCE
ICAM1	KNTC1	PDGFA
IDO1	KPNA2	PDGFRB
IFI27	LIG1	PDLIM7
IFIT2	LIN9	PLAT
IL15	MAD2L1	PMEPA1
IL17RB	MAX	RB1
IL2RA	MCM3	RUNX1
IL2RB	MCM5	SERPINF1
IL2RG	MCM6	SFRP4
IL6R	MELK	SPARC
IRF1	MKI67	SPDEF
IRF2	NASP	THBS1

IRF3
IRF4
IRF7
IRF9
ISG15
ITGB7
JAK2
LAG3
LYZ
MAP4K1
MX1
NFKB1
OAS1
OASL
PDCD1
PDCD1LG2
PML
PRDM1
PRF1
SLAMF7
SOCS1
STAT1
STAT2
TAP1
TAP2
TLR3
TNFAIP3
TRAF2
TYMP
VCAM1
NDC80
NOLC1
NUF2
NUP62
NUSAP1
PA2G4
PLK4
PML
POLE2
PRC1
PSIP1
RACGAP1
RAD9A
RPA1
RUVBL1
SKP2
SMC1A
SMC4
STMN1
TERF1
TFDP1
TOP2A
TOP3A
TP53
TPX2
TTK
UBB
WHSC1
THBS2
TIMP3
VEGFB
VEGFC
